# Mechanistic and functional characterization of NETs/IL-17 as a therapeutic target in EMT and brain metastasis of lung adenocarcinoma

**DOI:** 10.3389/fimmu.2026.1743841

**Published:** 2026-05-25

**Authors:** Yong Cai, Bin Su, Jiying Wang

**Affiliations:** 1 Department of Radiation Oncology, Shanghai Tianyou Hospital, Tongji University School of Medicine, Shanghai, China; 2 Department of Radiation Oncology, Shanghai Pulmonary Hospital, Tongji University School of Medicine, Shanghai, China; 3 Department of Oncology, Shanghai Pulmonary Hospital, Tongji University School of Medicine, Shanghai, China

**Keywords:** brain metastasis, epithelial-mesenchymal transition, H2BC4, lung cancer, neutrophil extracellular traps (NETs), IL-17A

## Abstract

**Background:**

Lung adenocarcinoma (LUAD) was known to have a propensity for brain metastasis. Neutrophil extracellular traps (NETs) played a role in facilitating tumor metastasis. However, the specific role of IL-17A in triggering the formation of NETs and its impact on NETs-mediated brain metastasis in LUAD was not well understood.

**Objective:**

We sought to investigate the IL-17A’s role in promoting NET formation, EMT, and brain metastasis in LUAD.

**Methods:**

We conducted LASSO and Cox regression analyses to pinpoint the essential genes in NETs that regulate LUAD. We carried out survival assays to validate the significance of the identified key genes. *In vitro* and *in vivo* models assessed NETs-IL17-induced EMT and metastasis, with P300’s role examined using siRNA and inhibitor B026. ChIP-qPCR and Co-IP revealed IL17A-DNA interactions and H2BC4/p300 recruitment to EMT gene promoters.

**Results:**

In neutrophils, IL-17A stimulation-induced NET formation may be associated with EP300-mediated increased acetylation of H2BC4. NETs activated IL17 signaling to drive EMT and brain metastasis. NETs enhanced tumor metastasis to brain, which was significantly suppressed by PAD inhibitors or DNase I. Furthermore, acetylated H2BC4, p300, c-Jun, and c-Fos were enriched at the promoter regions of CDH2, VIM, FN1, and ZEB1.

**Conclusion:**

Our research showed that IL-17A induced NET formation, and NETs-IL-17A stimulation were associated with increased H2BC4 expression and acetylation during the EMT process and brain metastasis of LUAD. These findings highlight a potential association between the IL-17A/NETs/H2BC4 pathway and the progression of brain metastasis, which may provide possible therapeutic targets for LUAD.

## 1 Introduction

Lung adenocarcinoma (LUAD) is the most frequent type of lung cancer, contributing to a significant number of cancer-related deaths globally (1). It is also the primary cause of brain metastases (BM) in patients with LUAD, as more than 30% of them develop BM (2–4). Diagnosis of BM typically occurs when patients present with headaches or neurological symptoms, leading to treatment options like surgery or radiotherapy for palliative care (5, 6). Unfortunately, patients with BM from LUAD typically have a survival rate of less than 7 months post-diagnosis (7). The rising incidence and death rates of BM necessitate the identification of potential drug targets, which requires a comprehensive understanding of LUAD brain metastases at both the cellular and molecular levels.

Neutrophils constitute the primary type of circulating granulocytes in humans, making up 50% to 75% of white blood cells in circulation (8). Their presence in the tumor microenvironment is crucial in regulating tumor progression (9, 10). Under infectious stimulation, neutrophils form NETs to capture and eliminate bacterial, fungal, and protozoan invaders, serving as a defense mechanism (11, 12). Researches have delved into the involvement of NETs in cancer, associating them with metastatic spread (13), cancer-related thrombosis (13), cancer immunoediting (14), progression (15, 16), and higher concentrations of NET markers in the plasma of cancer patients compared to healthy individuals (17). Additionally, NETs have been implicated in promoting cancer-related thrombosis in tumors, leading to a dismal prognosis for patients (15, 18). Observations of NETs in lung tissue, peripheral blood, and sputum of patients with lung cancer (19). suggest a potential role in brain metastasis by facilitating the early adhesion of tumor cells to distant organs through the capture of circulating tumor cells in severe postoperative sepsis. Neutrophils that are activated release neutrophil elastase (NE), potentially within NETs, which disrupts adherens junction proteins VE-cadherin and β-catenin and leads to an increase in BBB permeability (20). NETs can capture CTC in cases of severe postoperative sepsis, thereby promoting early adhesion of tumor cells to distant organ sites (21). This indicates that neutrophils may be involved in brain metastasis of lung cancer through NETs.

NETs are not only involved in capturing CTCS and disrupting BBBS, but they may also play a role in promoting brain metastasis of lung cancer cells through different mechanisms. They contain enzymes like NE, cathepsin G, myeloperoxidase (MPO), and nucleosomes, which consist of DNA and citrullinated histones (22). Additionally, IL-17A is present in high levels in NETs in patients with systemic lupus erythematosus, ankylosing spondylitis, and acute myocardial infarction (23–25). Studies in preclinical cancer models have shown that blocking IL-17 can inhibit metastasis, increase response to chemotherapy and radiotherapy (26–28). However, the impact of NETs carrying IL-17A on tumor cells within tumors has not yet been investigated.

This research will examine how NETs with IL-17A impact tumor cells. By using bioinformatics analysis, we identified H2BC4 as a key gene associated with NETs in LUAD tissues. As a result, we believe that H2BC4 could potentially have a significant role in the regulation of lung cancer cell behavior by NETs. Moving forward, our focus will be on studying the influence of NETs carrying IL-17A on the behavior of LUAD tumor cells and their role in promoting brain metastasis in lung cancer.

H2BC4, a member of the histone H2B family, is involved in the formation of chromosome nucleosomes and cell differentiation (29, 30). Limited research has been conducted on H2BC4, and there is no literature available on whether IL-17A signaling can impact its expression levels. H2BC4 is known to undergo modifications such as phosphorylation, methylation, and acetylation, with acetylation being the most common form in spermatogonia (31). The enzyme responsible for histone acetylation is the histone acetyltransferase (HAT) complex, which transfers acetyl groups from acetyl-CoA to lysine residues (32). p300 and its homolog, CBP, are key writers of lysine acetylation and are capable of acetylating NT lysine residues on all four histones (33–36). Studies have shown that p300 can mediate the acetylation of histone H2A-H2B (37). IL-17 has been shown to work in conjunction with IL-6 to enhance the recruitment of activated NF-κB p65, c-Fos, and c-Jun, as well as histone acetyltransferases CREB-binding protein and p300, to the IL-6 promoter to induce IL-6 transcription in astrocytes (38). Based on this information, it is possible to speculate that IL-17 in NETs may induce acetylation of H2BC4 by recruiting activated p300, potentially influencing the behavior of lung cancer cells and promoting brain metastasis in lung cancer.

What impact might NETs carrying IL-17A have on the behaviors of lung cancer cells that promote brain metastasis? The process of epithelial-mesenchymal transition (EMT) is crucial for tumor progression, invasion, and metastasis, as it allows cancer cells to become more invasive (39). NETs in LUAD have been found to be linked to EMT and angiogenesis, but only weakly associated with the cell cycle (40). Research has shown that NETs can trigger EMT in gastric and colorectal cancer cells (41, 42), with tumor-associated neutrophils producing IL-17a that promotes EMT in gastric cancer cells through JAK2/STAT3 signaling (43). IL-17 has also been found to induce quiescent gastric cancer stem cells to undergo EMT (44). The study will investigate if IL-17 in NETs induces H2BC4 acetylation via p300 to promote EMT in lung cancer cells, promoting brain metastasis. IL-17 is closely linked to the formation of NETs, as it enhances neutrophil infiltration and is a potent inducer of NETs formation (45–47). In the tumor microenvironment, IL-17A may stimulate the formation of NETs, though the specific mechanism requires further investigation.

Therefore, we will explore the mechanism by which IL-17A induces the formation of NETs. We will also investigate the impact of NETs carrying IL-17A on EMT and brain metastasis of tumor cells. This research will help identify new targets for screening targeted drugs for the treatment of brain metastases from lung cancer.

## 2 Materials and methods

### 2.1 Data collection and screening of target genes

In order to explore the key gene changes regulated by NETs in tumor cells, we obtained 69 NETs-initial biomarkers (40). Least absolute shrinkage and selection operator (LASSO) logistic regression analysis was used to screen out the key genes. Univariate Cox regression analysis was conducted to evaluate the effect of the genes on survival. The mRNA data of HPSE was obtained from TCGA and Clinical Proteomic Tumor Analysis Consortium (CPTAC) (https://proteomics.cancer.gov/programs/cptac), and the protein expression map of HPSE was derived from cProSite (https://cprosite.ccr.cancer.gov/). We collected data of the expression levels of H2BC4 in various cells from the HPA dataset and the Monaco dataset. The data from the TCGA database illustrated correlations in expression among the IL17A, IL17RA, H2BC4, and EP300 genes in LUAD. Overall survival and first progression survival analysis of LUAD patients was conducted using the Kaplan Meier plotter.

### 2.2 Cell culture

We obtained HL-60 cells (FH0101, FUHENG BIOLOGY) and H2122 cells (C6658, Beyotime). cells were cultured at 37 C in a humidified incubator with 5% CO_2_, using 1640 medium supplemented with 100 U/mL penicillin, 100 μg/mL streptomycin, and 10% fetal bovine serum.

### 2.3 Immunofluorescence analysis

The cells were fixed in 4% paraformaldehyde for 10 minutes, then permeabilized with 0.2% Triton X-100 in PBS for 10 minutes. Following two washes with PBS, cells were blocked with 5% FBS in PBS for 1 hour to reduce nonspecific antibody binding, and then incubated overnight at 4 C with anti-MPO (1:50, 66177-1-Ig, Proteintech) and anti-CitH3 (1:2000, ab281584, Abcam) primary antibodies. Subsequently, samples were treated with AF488-conjugated goat anti-rabbit (1:500, A0423, Beyotime) and AF555-conjugated donkey anti-mouse (1:50, A0460, Beyotime) secondary antibodies, mounted using an anti-fade sealing medium containing 4′,6-diamidino-2-phenylindole (DAPI, P0131-25ml, Beyotime), and visualized under a light microscope.

Paraffin-embedded animal tissue sections were dewaxed, rehydrated, subjected to antigen retrieval, blocked with bovine serum protein, and incubated overnight at 4 °C with anti-MPO (1:50, 66177-1-Ig, Proteintech), anti-CitH3 (1:2000, ab281584, Abcam), IL-17A (1:50, BF8019, Affinity), and IL-17RA (1:50, DF3602, Affinity) antibodies. After washing with PBS containing Tween 20 (PBST), sections were incubated at room temperature with AF488-conjugated goat anti-rabbit secondary antibody (1:500, A0423, Beyotime) and AF555-conjugated donkey anti-mouse (1:50, A0460, Beyotime) secondary antibodies, washed again with PBST, and counterstained using DAPI.

### 2.4 RT-qPCR

Then, proceed with collecting the cultured cells and lyse them with Trizol before sonication. Follow the RNA extraction steps, which involve chloroform extraction, centrifugation, ethanol precipitation, washing, and dissolution. Next, evaluate the concentration and purity of the RN. Subsequently, the extracted RNA is reverse transcribed into cDNA using a reaction mixture consisting of the RNA template, reverse transcriptase, primers, and dNTPs. After cDNA synthesis, utilize ChamQ Universal SYBR qPCR Master Mix (Q711-02, Vazyme) for qPCR analysis of gene expression levels. The primer sequences were listed in **Supplementary Table 1**. ACTB served as an internal control for quantifying mRNA levels, and the relative expression was calculated using the 2^-ΔΔCt^ method. The expression was normalized to ACTB levels. The experiments were repeated three times.

### 2.5 Western blotting

The cells were collected in pre-cooled centrifuge tubes. The lysis buffer (P0013, Beyotime) was added and lysis was performed on ice for approximately 30 minutes, mixing every few minutes by vortexing. The protein extract was obtained through centrifugation. A small portion of the protein extract was taken and the protein concentration was determined using the BCA protein assay kit (P0010, Beyotime). After the SDS-PAGE gel was prepared, the protein sample was mixed with the loading buffer in a specific ratio and heated in a boiling water bath for 5 minutes to denature the protein. The treated protein samples were loaded into the sample loading wells of the SDS-PAGE gel. PVDF membrane transfer was conducted. The blocking solution was removed, the diluted primary antibody was added, and incubated at 4 C overnight. The primary antibodies used were H2BC4 (1:1000, ab52484, Abcam), H2BK12Ac (1:2000, ab40883, Abcam), H2BK20Ac (1:500, ab240890, Abcam), p300 (1:1000, 20695-1-AP, Proteintech), Claudin 1 (1:30000, 28674-1-AP, Proteintech), EPCAM (1:1000, DF6311, Affinity), E-cadherin (1:50000, 20874-1-AP, Proteintech), N-Cadherin (1:8000, 22018-1-AP, Proteintech), Vimentin (1:5000, 10366-1-AP, Proteintech), Fibronectin (1:1000, CY9537, Abways), ZEB1 (1:1000, DF7414, Affinity), Slug (1:1000, AF4002, Affinity), Snail(1:1000, CY3066, Abways), p-SMAD2 (1:1000, AF3367, Affinity), SMAD2 (1:1000, AF6367, Affinity), IκBα (1:20000, 10268-1-AP, Proteintech), p-p65^Ser536^ (1:1000, AF5006, Affinity), p65 (1:1000, AF2006, Affinity), p-p38 (1:1000, AF3455, Affinity), p38 (1:2000, AF6456, Affinity), Tubulin (1:300000, T0023, Affinity) and actin (1:150000, T0022, Affinity). Subsequently, the secondary antibody was incubated with the samples. The experiments were repeated three times.

### 2.6 Cell transfection

For EP300 knockdown, the short hairpin RNAs (siRNA) and a non-targeting RNA (scrambled RNA) were designed and synthesized. The siRNA sequences were 5’-AUUCCGAGACAUCUUGAGATT-3’ (siEP300-1); 5’-CUUCACAAUUCCGAGACAUTT-3’ (siEP300-2); 5’-CGGUGAACUCUCCUAUAAUTT-3’ (siEP300-3). When the cell confluence reached approximately 50%, 1.5 μL of the siRNA-Mate plus transfection reagent (G04026, GenePharma) was added to prepare the siRNA pre-mix solution. The solution was then immediately mixed thoroughly using a pipette. Subsequently, the mixture was added dropwise to the cells in a 24-well plate, and the plate was gently shaken to ensure even distribution. After application, the plate was returned to the incubator for cell culture. The expression level was assessed 48 hours after transfection.

### 2.7 Chromatin immunoprecipitation analysis

Cells were grown in 150mm culture dishes until reaching around 90% confluence. At that stage, the medium was replaced with fresh medium containing 1% formaldehyde to initiate cross-linking. The cells were fixed at room temperature for 10 minutes. To neutralize excess formaldehyde and halt cross-linking, 2ml of 10× glycine was gently added to each dish and mixed thoroughly. The cells were incubated for an additional 5 minutes at room temperature. Afterwards, the cells were washed with PBS, then 2ml of 1× PBS with a protein inhibitor cocktail was added. Cells were harvested and resuspended in 0.5ml of lysis buffer containing the same inhibitor cocktail. The cell suspension was kept on ice for 15 minutes, with gentle vortexing every 5 minutes, then the process was repeated once more for comprehensive lysis. The supernatant obtained was sonicated to fragment the DNA. The lysate was then centrifuged at 12,000g for 10 minutes at 4 C. Post-centrifugation, 50 μL of supernatant (lysate) was transferred into a new microtube as the DNA input for immunoprecipitation. To this, 450 μL of pre-prepared dilution buffer was added and mixed well by vortexing. An appropriate amount of antibody or IgG (1:50 dilution) was added to each tube, followed by incubation on a rotator at 4 C for at least 4 hours or overnight for immunoprecipitation. Next, 20 μL of protein A/G magnetic beads were added to each tube and rotated at 4 C for 2 hours to capture the antibody–protein/DNA complexes. The bead-bound complexes were washed following the manufacturer’s protocol. ChIP elution buffer containing proteinase K was used to elute the complexes, with incubation at 62 C for 2 hours. Samples were then heated at 95 C for 10 minutes, and allowed to cool to room temperature. The tubes were centrifuged at 10,000g for 10 seconds, and any material adhering to the tube lids or sides was collected using a magnetic stand. The supernatant was carefully transferred into a new tube, and DNA purification was performed according to the kit instructions. Finally, the purified DNA was subjected to qPCR analysis. All ChIP enrichment levels are shown relative to Veh. group, and IgG and Input were used as the negative control and positive control, respectively. The experiments were repeated three times.

### 2.8 NET isolation

HL-60 cells were first differentiated into neutrophil-like cells by resuspending at a density of 2.5 × 10^5^ cells/mL in RPMI-1640 complete medium supplemented with 1.25% DMSO in 15-cm culture dishes, followed by incubation for 5 days at 37 C and 5% CO_2_. Differentiated cells were then harvested, washed once with PBS, and resuspended in RPMI-1640 medium (without serum) supplemented with 500 nM phorbol-12-myristate-13-acetate (PMA) at a final concentration of 5 × 10^6^ cells/mL. For NET induction, 30 mL of the cell suspension (total 1.5 × 10^8^ cells) was seeded in each 15-cm dish and incubated for 4 hours at 37 C.

Following induction, the supernatant was carefully removed, and the dish was gently washed once with PBS to eliminate non-adherent cells and residual medium. NETs, together with adherent cells, were then scraped from the plate in fresh PBS and collected. The resulting suspension was centrifuged at 300g for 10 minutes at room temperature to pellet intact cells and debris. The supernatant, containing soluble NETs, was carefully collected and used for subsequent experiments or analysis. NET concentrations were approximated quantified by measuring the DNA content in the supernatant with a microplate reader.

### 2.9 RNA sequencing and Bioinformatics analysis

RNA sequencing of H2122 cells treated with NETs was conducted by Beijing Novogene Co., Ltd. Total RNA was extracted from lung tissue using Trizol reagent, and both RNA quality and quantity were assessed. After cDNA synthesis, the quality of the cDNA library was evaluated using the Agilent 2100 bioanalyzer. Sequencing was then performed on an Illumina platform, where the fluorescence signals from each cluster were captured and translated into corresponding bases, yielding the sequencing data.

Raw sequencing reads were subjected to quality control using fastp (v0.20.1) to remove adaptors and low-quality reads. Clean reads were then aligned to the reference genome using HISAT2 (v2.2.1). Gene-level quantification was performed using featureCounts (v2.0.1). Expression data were normalized, and differential gene expression analysis was conducted using the DESeq2 package (v1.34.0) in R. Differentially expressed genes (DEGs) were defined as those with an adjusted p value (Padj) < 0.05, as determined by the Benjamini and Hochberg’s approach for multiple testing correction, and with |log2FoldChange| > 1.

Subsequent functional annotation of DEGs, including gene ontology (GO) enrichment and Kyoto Encyclopedia of Genes and Genomes (KEGG) pathway analysis, was performed using the clusterProfiler package (v4.2.2) in R.

### 2.10 Immunoprecipitation analysis

The extracted NETs were fragmented into 200–500 bp DNA fragments using an ultrasonic cell disruptor. To the enzymatic reaction solution, three volumes of P3 Buffer and one volume of isopropanol were added, and the mixture was vortexed thoroughly. DNA purification was then performed according to the protocol provided with the kit. A DNA template (100 ng to 1 μg) was adjusted to a final volume of 34 μL with an appropriate amount of ultrapure water. Subsequently, 10 μL of Random Primer in Buffer (5X) was added. The solution was mixed well and heated in boiling water for 5 minutes, followed by immediate cooling in an ice-water bath for 3 minutes. Next, 5 μL of Biotin-Labeling Mix was added, and the mixture was gently mixed. Afterward, 1 μL of Klenow Fragment was introduced, and the reaction mixture was incubated overnight at 37 C. To terminate the labeling reaction, 3 μL of probe labeling termination solution was added and mixed thoroughly. The dHL-60 cells treated with PMA for 4 hours were collected. The cells were lysed by pipetting on ice in 400 μL of RIPA lysis buffer containing 1 mM PMSF. The lysate was then incubated with magnetic beads coupled to biotin-labeled NETs-DNA, and the mixture was rotated overnight at 4 C to facilitate binding. After incubation, the magnetic beads were washed thoroughly. To elute the bound complexes, 100 μL of Acid Elution Buffer was added, the mixture was incubated at room temperature for 5 minutes, and then separated. Finally, the supernatant was transferred to a new centrifuge tube and used as the sample for WB analysis.

### 2.11 Pull-down experiment

Streptavidin magnetic beads (40 μL) were placed into an RNase-free EP tube. The beads were washed by adding 1 mL of 1× TES buffer. The tube was then set on a magnetic rack for 1 minute to allow the beads to separate from the supernatant, which was carefully discarded. Next, 1 μg of biotin-labeled NETs-DNA, an appropriate volume of 2× TES buffer, and the washed magnetic beads were added to a new tube. The reaction was gently mixed at 25 C for 30 minutes on a rotator to allow binding. Following incubation, the magnetic beads were washed once more to obtain the NETs-DNA–magnetic bead complex. In a separate RNase-free EP tube, 50 nM recombinant IL17A protein was added, followed by 5 μL of DNase and 2.5 μL of DNase salt stock. The mixture was gently incubated at 25 C for 1 hour. After incubation, 40 μL of agarose beads were added and the tube was rotated gently at 4 C for 30 minutes. The mixture was centrifuged at 3000g for 1 minute at room temperature. The resulting supernatant was carefully transferred to a new EP tube. To the collected supernatant, 500 μL of Binding buffer, 9 μL of EDTA, 4.5 μL of EGTA, 5 μL of protease inhibitor, 5 μL of DTT, and 5 μL of poly(dI·dC) were added and the mixture was thoroughly mixed to ensure a homogeneous solution. The previously prepared NETs-DNA–magnetic bead complex was then incorporated into this mixture. For the control (input) group, another RNase-free EP tube was prepared and 50 nM recombinant IL17A protein was added, along with the same amount of magnetic beads as initially used. This mixture was incubated by rotation at 4 C for 1 hour as well. After incubation, the magnetic beads in both experimental and control groups were washed. Then, 60 μL of Protein Elution buffer and 0.6 μL of DTT were added to each tube. The tubes were incubated at 37 °C for 2 hours, with intermittent mixing. Finally, the tubes were placed on a magnetic rack for 1 minute to collect the magnetic beads, and the supernatant was transferred to new EP tubes for WB analysis. The experiments were repeated three times.

### 2.12 Establishment of subcutaneous tumor-bearing mice model and metastasis mice model

Nude mice were randomly assigned to experimental groups using a random number generator. For each experimental group, N = (6) mice were used unless otherwise specified. The experimental groups included: vehicle, NETs, NETs + PAD4 inhibitor, NETs + DNase I, IgG, NETs + IL-17A antibody, and NETs + IL-17RA antibody groups. Each mouse was subcutaneously inoculated with 5×10^6^ H2122 cells. When the tumor diameter reached approximately 0.7cm, the following treatments were administered: in the NETs group, 2 μg of NETs were injected directly into the tumor; in the NETs + PAD4 inhibitor group, GSK484 was administered intraperitoneally at a concentration of 20 mg/kg; in the NETs + DNase I group, DNase I (300 U/mouse) was mixed with NETs and injected into the tumor; and in the NETs + IL-17A antibody and NETs + IL-17RA antibody groups, the respective antibodies (10 ng/mouse) were injected intravenously. The IgG group received an equal volume of IgG, and the vehicle group was injected with an equal volume of PBS, either into the tumor or intravenously, corresponding to the respective control treatments.

H2122 cells were used to establish a mouse model of lung cancer metastasis by tail vein injection. Initially, the cells were digested, counted, and then resuspended in pre-cooled PBS to achieve a concentration of 1×10^7^ cells/mL. A total of 100 μL of this cell suspension, containing 1×10^6^ cells, was injected into each nude mouse via the tail vein. The mice were randomly assigned to experimental groups using a random number generator. For each experimental group, N = (6) mice were used unless otherwise specified. The experimental groups included: vehicle, NETs, NETs + PAD4 inhibitor, NETs + DNase I, NETs + IL17A antibody, and NETs + IL17RA antibody. The vehicle group received the same volume of PBS as a control. For the treatment groups, NETs were administered at a dose of 20 μg/100 μL per mouse, GSK484 (PAD4 inhibitor) at 20 mg/kg, DNase I at 300 U/mouse, and IL17A and IL17RA antibodies at 10 ng/mouse each. Six weeks after the injections, *in vivo* imaging was conducted to assess metastasis. Subsequently, mice from the vehicle and NETs groups were sacrificed, and their liver and brain tissues were fixed and sectioned for hematoxylin & eosin (H&E) staining and immunofluorescence analysis. All animal experiments were approved by the Animal Ethics Committee of Tongji University School of Medicine.

### 2.13 H&E staining

Brain and liver tissues pathology were examined using hematoxylin & eosin (H&E). The slices for were treated with Harris hematoxylin (G1004, Servicebio) for 8min, eosin (G1002, Servicebio) dye for 3min, and then sealed with neutral gum. A microscope was used to observe the staining results.

### 2.14 Cellular thermal shift assay analysis

Cells were seeded in 6-well plates and incubated until approximately 80% confluence was reached. B026 was added to the wells at final concentrations of 0, 0.25, 0.5, 1.0, and 2.0 μM, respectively. The cells were then incubated at 37 C for 2 hours. Following incubation, the cells were digested with trypsin. Trypsin was neutralized by adding complete medium, and the cells were collected by centrifugation. The supernatant was discarded, and the cell pellet was resuspended in 100 μL PBS. The resuspended cells were placed in a 50 C water bath for 3 minutes and then immediately transferred to liquid nitrogen for rapid cooling. RIPA lysis buffer was added to lyse the cells, and the lysate was centrifuged at 13,000g. The supernatant was collected as the protein solution for subsequent WB analysis. The experiments were repeated three times.

### 2.15 Statistical analysis

The hTFtarge (https://guolab.wchscu.cn/hTFtarget#!/) was used to predict the targeting properties of c-Jun and c-Fos for the EMT related genes that were targeted for EMT. The data in the study was presented using means ± standard deviation (SD). GraphPad Prism 8.0 from GraphPad Software, USA, was used to analyze group differences. Two group comparisons were conducted using Student’s t-tests, while multiple group comparisons were made with one-way analysis of variance followed by Tukey’s *post-hoc* test. Statistical significance was indicated by *P* values < 0.05.

## 3 Results

### 3.1 H2BC4 was key gene in the regulation of LUAD by NETs

**Figure 1:**
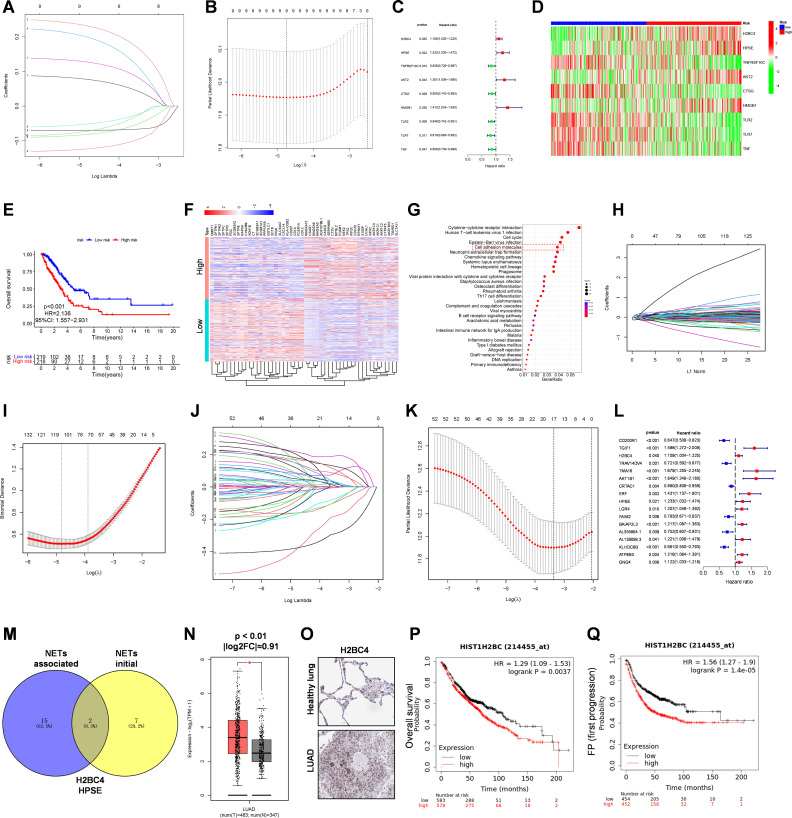
Signature genes of NETs and its associated signals in LUAD. **(A, B)**. Coefficients and partial likelihood deviance in LASSO regression analysis of 69 NETs-initial biomarkers. **(C)** Univariate COX regression analysis of 9 prognostic NETs-initial genes. **(D)** Expression profile of 9 prognostic NETs-initial genes in high-NETs score and low-NETs score groups. **(E)** Survival analysis between high-NETs score and low-NETs score groups. **(F)** Top 50 differentially expressed genes (DEGs, in total 3794) between high-NETs score and low-NETs score groups. **(G)** KEGG pathway enrichment analysis based on 3794 DEGs (NETs associated genes). **(H, I)** Coefficients and partial likelihood deviance in LASSO regression analysis of all genes (n = 3794) in NETs’ associated signals; dependent variable is NETs score. **(J, K)** Coefficients and partial likelihood deviance in LASSO regression analysis of NETs associated signature genes. **(L)** Univariate COX regression analysis of 17 prognostic NETs associated signature genes. **(M)** Overlapping between NETs initial and NETs associated genes. **(N)** H2BC4 mRNA levels in LUAD samples compared with non-tumor lung tissues. **(O)** H2BC4 protein levels detected by IHC in LUAD samples compared with healthy lung tissues from THPA (https://www.proteinatlas.org/). **(P, Q)** Overall survival and first progression survival analysis of LUAD based on H2BC4 expression level using Kaplan-Meier Plotter (https://kmplot.com/analysis/index.php).

To explore the changes in which key genes of tumor cells are regulated by NETs, we conducted least absolute shrinkage and selection operator (LASSO) logistic regression analyses on 69 NETs-initial biomarkers (with survival status and survival time as the dependent variables) to identify 9 NETs characteristic genes in LUAD (**Figures 1A, B**). Then, we performed Univariate COX regression analysis on these 9 genes, and the results indicated that all of them had independent prognostic capabilities in LUAD (**Figure 1C**). Based on these findings, we scored LUAD samples obtained from TCGA database using these 9 genes and divided them into high-NETs score and low-NETs score groups (**Figure 1D**). Subsequently, a survival analysis was conducted between the high-NETs score and low-NETs score groups, revealing that high NETs were associated with a worse prognosis (**Figure 1E**). Further analysis involved examining differentially expressed genes (DEGs) in these two groups, which led to the identification of 3,794 DEGs. The top 50 DEGs are presented in **Figure 1F**.

Next, we conducted a KEGG analysis on these 3794 DEGs. The results showed that the DEGs were mainly enriched in the pathways related to Cytokine-cytokine receptor interaction, Human T-cell leukemia virus 1 infection, and Cell adhesion molecules (**Figure 1G**). We performed LASSO regression analysis on 3,794 differentially expressed genes between the high and low NETs score groups, using the NETs score as the dependent variable. Through this analysis, 109 related genes were identified (**Figures 1H, I**). Then, we subjected these 109 genes to univariate Cox regression analysis and selected those with p < 0.05, resulting in 53 NETs associated signature genes (**Figures 1J, K**). Finally, another round of LASSO regression was conducted, this time with survival status and survival time as dependent variables, to further screen for NETs signature genes (17) that were strongly associated with prognosis (**Figure 1L**). We conducted an intersection of NETs-initial genes and NETs-associated signature genes to identify two key genes, H2BC4 and HPSE (**Figure 1M**). The HPSE mRNA is upregulated in LUAD while the protein is downregulated (**Supplementary Figure 1**). In LUAD samples, the H2BC4 mRNA levels are higher compared to non-tumor lung tissues (**Figure 1N**), and the IHC results showed that the H2BC4 protein level in LUAD lung tissues was higher than in healthy lung tissues (**Figure 1O**). The Kaplan-Meier survival analysis revealed that high expression of H2BC4 was associated with poor overall survival (OS) and first progression in LUAD patients (**Figures 1P, Q**). Therefore, we will focus on using H2BC4 as the key gene for research to investigate the role of H2BC4 in the impact of NETs on LUAD cells.

Moreover, in addition to tumor cells, we also compared the expression of H2BC4 in various cells, with results showing that H2BC4 was expressed at the highest level in neutrophils, indicating a certain specificity (**Supplementary Figure 2**). Hence, we would further investigate whether H2BC4 regulated the production of NETs in neutrophils and elucidated the underlying mechanism. Subsequently, we would explore whether p300 mediates the regulation of IL17 on the expression level of H2BC4. So, we analyzed the correlations of the expression of IL17A, IL17RA, H2BC4, and EP300 genes in LUAD. Our analysis revealed that the expression of IL17A positively correlated with the expressions of IL17RA, H2BC4, and EP300. Additionally, the expression of IL17RA positively correlated with the expressions of H2BC4 and EP300. Moreover, the expression of H2BC4 positively correlated with the expression of p300 (**Supplementary Figure 3**).

### 3.2 The upregulation of H2BC4 expression and acetylation in neutrophils played a role in the formation of NETs triggered by IL17A

**Figure 2:**
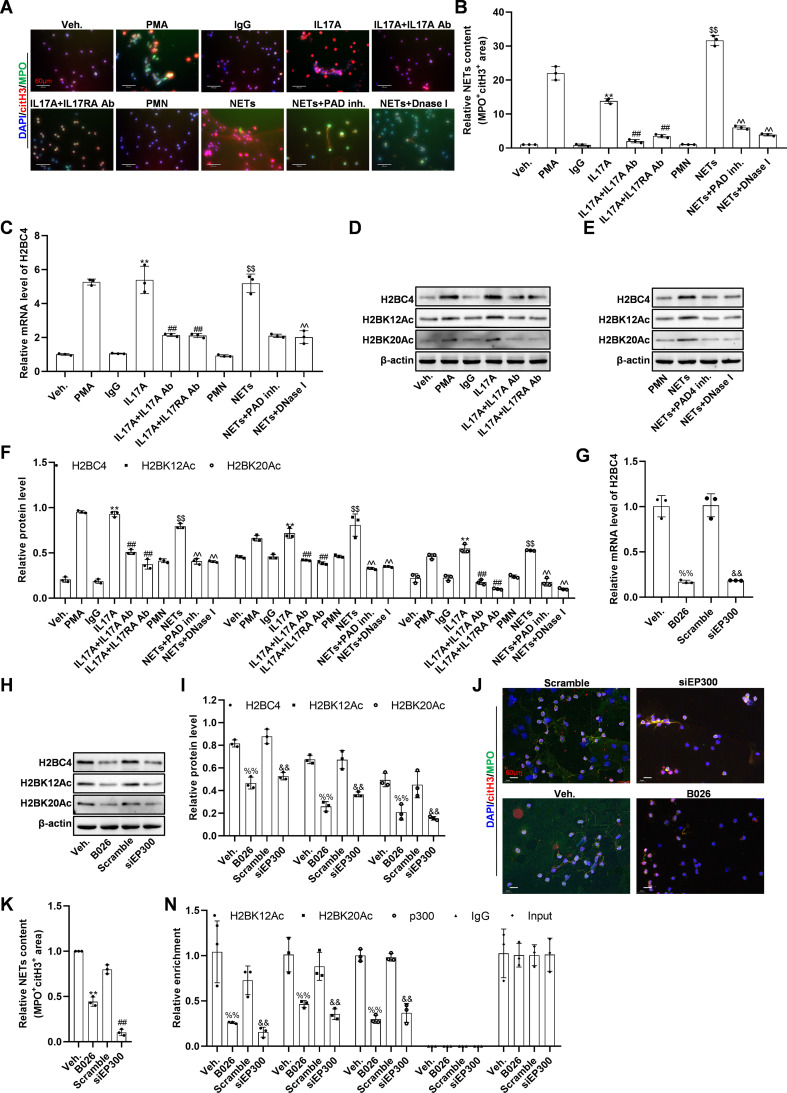
The increased H2BC4 expression and acetylation in neutrophils were involved in IL17A-mediated NETs formation *in vitro*. **(A, B)** Immunofluorescence double labeling was used to detect NETs formation of IL17A-treated neutrophils. **(C)** RT-qPCR was used to detects the H2BC4 expression in IL17A-treated neutrophils. **(D-F)** Western blotting detects H2BC4 protein, acetylation of H2BC4 (H2BK12Ac, H2BK20Ac) in IL17A-treated neutrophils. **(G-I**) Cells treated with IL-17A were simultaneously treated with inhibitors or siRNA to interfere with Ep300, and western blotting was used to detect the levels of H2BC4 mRNA, H2BC4 protein and H2BC4 acetylation. **(J, K)** Immunofluorescence double labeling was used to measure the effects of inhibiting p300 on PMA-induced NETs formation. **(L, M)** Immunofluorescence double labeling was used to detect the effect of inhibiting p300 on IL17A-induced NETs formation. **(N)** ChIP-qPCR was used to determine the enrichment of H2BK12Ac, H2BK20Ac, and p300 in the H2BC4 promoter region of IL17A-induced neutrophils. All ChIP enrichment levels are shown relative to Veh. group, and IgG and Input were used as the negative control and positive control, respectively. The experiments were repeated three times (n=3). ^**^P < 0.01, compared with IgG group; ^##^*P* < 0.01, compared with IL17A group; ^$$^*P* < 0.01, compared with PMN group; ^^^^*P* < 0.01, compared with NETs group.

Firstly, we investigated the influence of H2BC4 on IL17A-mediated NETs formation. Immunofluorescence results revealed that PMA and IL17A both increased NETs concentration, while anti-IL17A and anti-IL17RA antibodies mitigated the effect of IL17A (**Figures 2A, B**). When neutrophils were treated with NETs, there was an increase in NETs formation in neutrophils, with PAD inhibitors and DNase I counteracting the effect of NETs. We measured the H2BC4 expression in neutrophils post treatment with IL17A and NETs. The outcomes demonstrated that PMA and IL17A boosted mRNA and protein levels of H2BC4, as well as protein levels of H2BK12Ac and H2BK20Ac in neutrophils (**Figures 2C-F**), whereas anti-IL17A and anti-IL17RA antibodies attenuated the effect of IL17A. Furthermore, NETs triggered a rise in mRNA and protein levels of H2BC4, as well as protein levels of H2BK12Ac and H2BK20Ac in neutrophils, with PAD inhibitors and DNase I mitigating the impact of NETs.

To delve into the impact of p300 on H2BC4 acetylation, neutrophils were treated with EP300 siRNA and the small molecule inhibitor B026 to decrease EP300/p300 expression. EP300 siRNA notably decreased mRNA and protein levels, with siEP300#1 showcasing the highest interference efficiency and thus utilized as siEP300 in subsequent experiments (**Supplementary Figures 4A-C**). The cellular thermal shift assay illustrated that small molecule inhibitor B026 decreased EP300 protein levels in a dose-dependent manner (**Supplementary Figures 4D, E**).

Neutrophils were exposed to IL17A and the B026 inhibitor or EP300 siRNA to further evaluate the impact. Both the B026 inhibitor and EP300 siRNA lowered H2BC4 mRNA levels and protein levels of H2BC4, H2BK12Ac, and H2BK20Ac (**Figures 2G-I**). Subsequently, both the B026 inhibitor and EP300 siRNA diminished levels of NETs induced by IL17A (**Figure 2J**). Furthermore, ChIP experiments displayed that the H2BC4 promoter region in neutrophils was enriched with H2BK12Ac, H2BK20Ac, and p300 during IL17A-induced NETs (**Figure 2N**). These findings suggest that p300 probably play a crucial role in regulating H2BC4 acetylation and expression levels in neutrophil NETs formation induced by IL17A.

### 3.3 NETs promoted EMT and brain metastasis by activating IL-17 pathway in tumor

**Figure 3:**
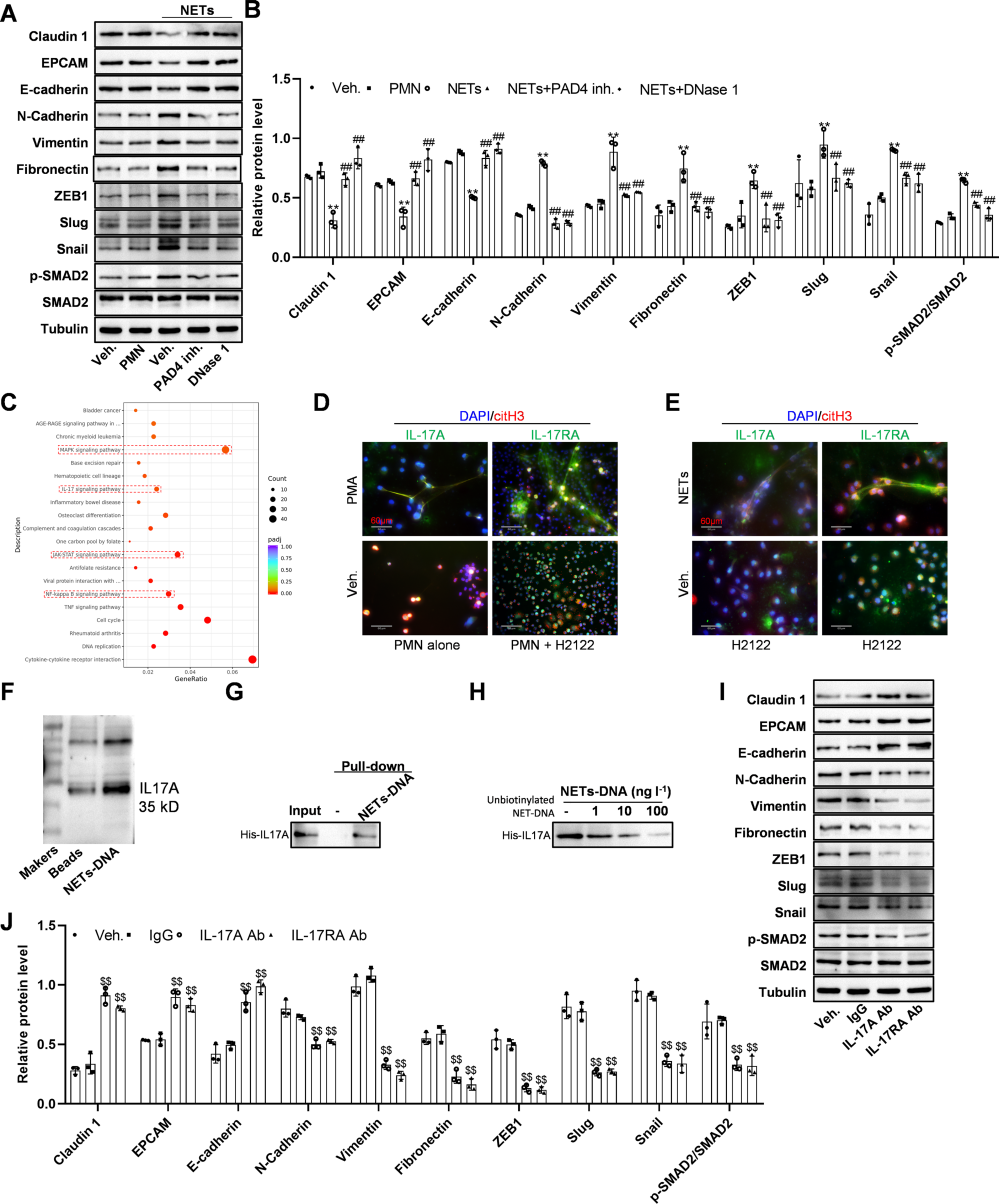
NETs promoted EMT by activating the IL-17 pathway in H2122 cells. **(A, B)** Western blot analysis of the protein expression levels of EMT markers after NETs treatment in H2122 cells. **(C)** KEGG pathway enrichment analysis of DEGs screened by transcriptome sequencing after NETs treatment in H2122 cells. **(D)** Double fluorescence labeling was used to detect the co-localization of IL17A on NETs or IL17RA on H2122 cells *in vitro* co-culture of H2122 cells and PMN cells. **(E)** Double fluorescence labeling was used to detect the co-localization of IL17A on NETs or IL17RA on H2122 cells in H2122 cells treated with NETs. **(F)** Immunoprecipitation was used to detect the presence of IL17A in NETs. Beads are unbound, and NET-DNA is connected with biotinylated NET-DNA. **(G)** Pull-down experiment to verify the interaction between IL17A and DNA on NETs. **(H)** Repeat of the Pull-down experiment mentioned above in the presence of non-biotinylated NET-DNA **(I, J)**. Following the application of NETs and IL17A or IL17RA neutralizing antibodies in the H2122 cells, the expression of EMT markers was measured. The experiments were repeated three times (n=3). ^**^P < 0.01, compared with PMN group; ^##^*P* < 0.01, compared with NETs group; ^$$^*P* < 0.01, compared with IgG group.

Next, we investigated the effects of NETs/IL-17 on tumor cell EMT and brain metastasis. Firstly, we explored the effect of NETs on the EMT of H2122 cells. The results showed that NETs reduced the levels of Claudin 1, EPCAM, and E-cadherin in H2122 cells, and increased the levels of N-Cadherin, Vimentin, Fibronectin, ZEB1, Slug, Snail, and p-SMAD2/SMAD2 (**Figures 3A, B**). The PAD inhibitor and DNase I reduced the effects of NETs.

To further explore the key signaling pathways regulated by NETs in H2122 cells, we performed transcriptome sequencing after treating H2122 cells with NETs, and then conducted bioinformatics analysis. The DEGs were discriminated using a cutoff value of *P*adj < 0.05 and |Log2foldchange| > 1. Compared with the Sham group, a total of 589 downregulated and 1023 upregulated DEGs were identified in NETs-treated H2122 cells (**Supplementary Figure 5**). According to the GO annotations, the 1612 DEGs were primarily involved in the biological processes related to response to stress. The majority of the annotations for cellular components were concentrated in the extracellular region and extracellular region part. Regarding molecular functions, molecular function regulator and signaling receptor binding were mainly enriched (**Supplementary Figure 5**). The differentially expressed genes were subjected to KEGG pathway enrichment analysis. The results showed that the main pathways enriched by the differentially expressed genes were MAPK signaling pathway, IL-17 signaling pathway, JAK-STAT signaling pathway, and NF-kappa B signaling pathway (**Figure 3C**). Studies have shown that IL-17 may be present in NETs. Therefore, we will subsequently explore whether NETs regulate the EMT of H2122 cells through IL-17 and the mechanism involved.

The neutrophils treated with PMA (containing NETs and neutrophils) or NETs isolated after PMA treatment of neutrophils were used to treat H2122 cells. Then, the co-localization of IL17A on NETs or IL17RA on H2122 cells and citH3 in the co-culture system was detected by immunofluorescence. The results showed that IL17A on NETs and IL17RA on H2122 cells could all co-localize with citH3 (**Figures 3D, E)**. Then, we determined whether there was IL17 in NETs through IP experiments. The results showed that there was IL17A in NETs (**Figure 3F**). Additionally, the Pull-down experiment results indicated that IL17 was present in NETs and interacted with DNA (**Figures 3G, H**). Subsequently, we explored whether IL17 regulates the EMT of H2122 cells. The results showed that the neutralizing antibodies against IL17A and IL17RA increased the levels of Claudin 1, EPCAM, and E-cadherin, while reducing the levels of N-Cadherin, Vimentin, Fibronectin, ZEB1, Slug, Snail, and p-SMAD2/SMAD2 (**Figures 3I, J**).

**Figure 4:**
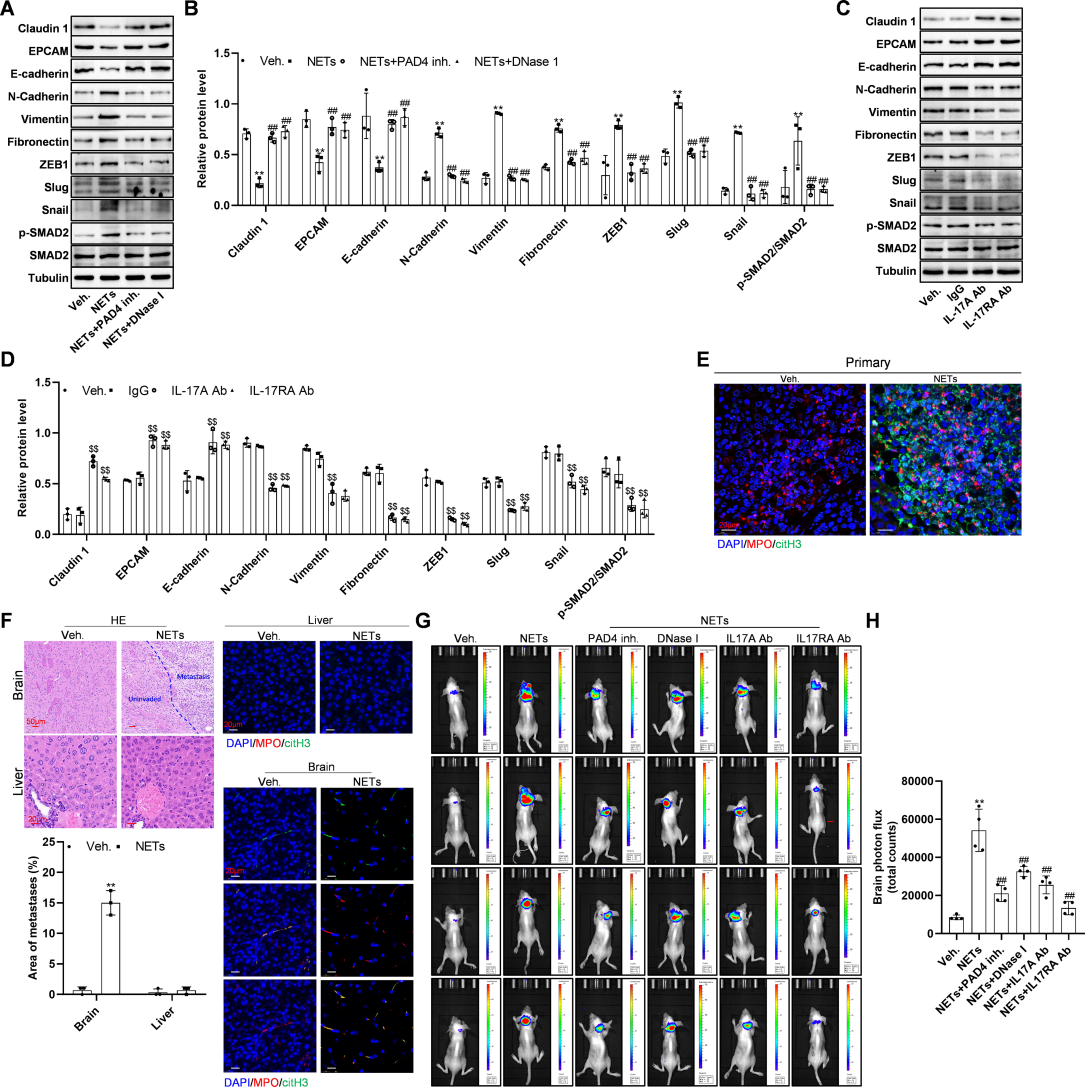
NETs promoted EMT and brain metastasis by activating IL-17 pathway in tumor. **(A, B)**. After injecting NETs into the tumor masses of subcutaneously implanted mice, WB was used to detect the EMT markers in the tumor tissue. **(C, D)**. After treating subcutaneously implanted mice with IL17A and IL17RA neutralizing antibodies, the impact on the EMT phenotype was detected using WB. **(E)**. Dual fluorescence labeling was used to detect the NETs in the lung cancer tissues (N = 4). **(F)**. In the metastasis model, HE staining and immunofluorescence double labeling were used to characterize the cancer cell metastasis and detect the levels of NETs in the brain and liver metastatic tissues (N = 4). **(G, H)**. *In vivo* biological imaging was employed to assess the effects of the PAD4 inhibitor, DNase I, IL17A neutralizing antibody, and IL17RA neutralizing antibody on NETs-induced brain metastasis tissues of LUAD (N = 4). The WB experiment was repeated three times (n=3). ^**^P < 0.05, compared with Veh. group; ^##^*P* < 0.01, compared with NETs group; ^$$^*P* < 0.05, compared with IgG group.

We treated lung cancer mice with NETs (subcutaneous tumor-bearing model) and measured the EMT markers in lung cancer tissues. The results showed that NETs decreased the levels of Claudin 1, EPCAM, and E-cadherin, and increased the levels of N-Cadherin, Vimentin, Fibronectin, ZEB1, Slug, Snail, and p-SMAD2/SMAD2 (**Figures 4A, B**). Moreover, the neutralizing antibodies against IL17A and IL17RA increased the levels of Claudin 1, EPCAM, and E-cadherin in tumor tissues (subcutaneous tumor-bearing model) and decreased the levels of N-Cadherin, Vimentin, Fibronectin, ZEB1, Slug, Snail, and p-SMAD2/SMAD2 (**Figures 4C, D**). Immunofluorescence was used to determine the levels of NETs in lung cancer mice treated with NETs in lung cancer tumor tissues. The results showed that NETs treatment increased the levels of NETs (**Figure 4E**). Next, we established a metastatic tumor model, and then detected the levels of NETs in the brain and liver tissues. The HE staining results showed that NETs promoted the lung cancer cells to spread to the brain. But neither the NETs nor the control group’s lung cancer cells showed liver metastasis. The NETs levels were higher in the brain metastasis lesions of the NETs treatment group (**Figure 4F**). In addition, *in vivo* biological imaging results also showed that NETs promoted tumor cell brain metastasis, while the PAD inhibitor and DNase I both inhibited the effect of NETs, and the neutralizing antibodies against IL17A and IL17RA decreased the effect of NETs (**Figures 4G**, **3H**).

### 3.4 The NETs-IL-17 signal activated EP300-mediated H2BC4 expression and acetylation to regulate the EMT of H2122 cell

**Figure 5:**
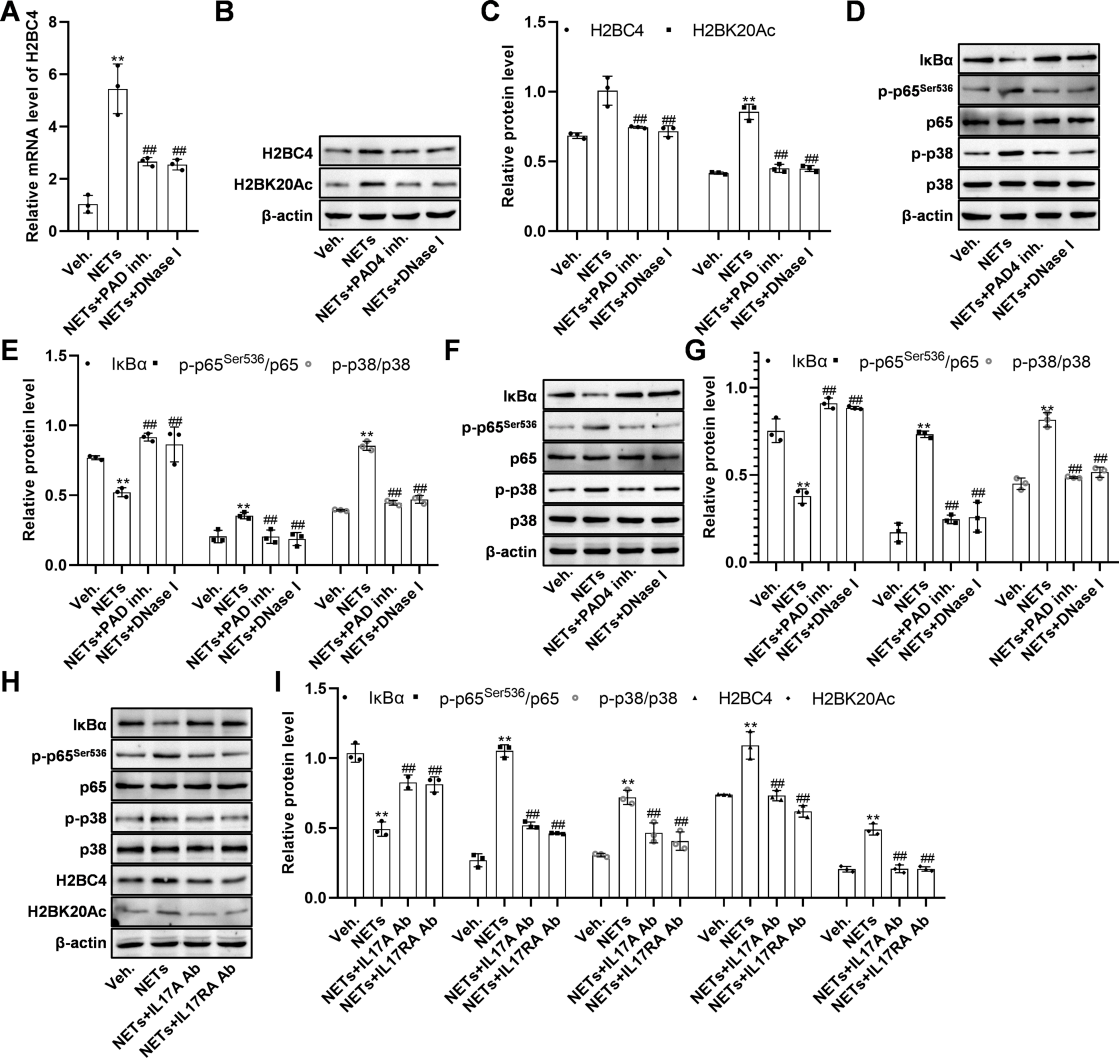
NETs activated p300-mediated H2BC4 expression and acetylation through IL-17 signal in LUAD. **(A)**. The effects of the PAD4 inhibitor, DNase I on H2BC4 mRNA in H2122 cells treated with NETs. **(B, C)**. The effects of the PAD4 inhibitor, DNase I on H2BC4 protein and acetylated H2BC4 protein levels in H2122 cells treated with NETs. **(D, E)**. The effects of the PAD4 inhibitor, DNase I on the levels of IL17 signaling pathway-related molecules in H2122 cells treated with NETs. **(F, G)**. The effects of PAD4 inhibitor and DNase I on the levels of IL17 signaling pathway-related molecules in subcutaneous tumor-bearing mice treated with NETs. **(H, I)**. The effects of IL17A, IL17RA neutralizing antibodies on the levels of IL17 signaling pathway-related molecules, H2BC4 protein, and acetylated H2BC4 protein in subcutaneous tumor-bearing mice treated with NETs. The cell experiment and WB experiment were repeated three times (n=3). ^**^P < 0.05, compared with Veh. group; ^##^*P* < 0.01, compared with NETs group.

We investigated the effect of NETs on the acetylation of H2BC4 in H2122 cells. The results showed that NETs induced an increase in the mRNA level of H2BC4 in H2122 cells, as well as an increase in the levels of H2BC4 protein and acetylated H2BC4 protein (H2BK20Ac). The PAD inhibitor and DNase I both inhibited the effect of NETs (**Figures 5A-C**).

Further, the levels of molecules related to the IL17 signaling pathway were measured. The results showed that NETs reduced the level of IκBα in H2122 cells, and increased the levels of p-p65Ser536/p65 and p-p38/p38 (**Figures 5D, E**). In subcutaneous tumor-bearing mouse tissues, NETs treatment also reduced the level of IκBα in tumor cells, and increased the levels of p-p65Ser536/p65 and p-p38/p38 (**Figures 5F, G**). The PAD inhibitor and DNase I both inhibited the effect of NETs. Additionally, the IL17A and IL17RA neutralizing antibodies also reduced the effect of NETs (**Figures 5H, I**).

Next, to investigate which EMT-related genes expressed by tumor cells might be affected by NETs and whether p300 is involved in mediating this regulation, we used hTFtarget to predict whether c-Jun and c-Fos can target key EMT related genes. The results showed that both c-Jun and c-Fos are able to target the VIM gene, which promotes EMT. However, among the other EMT-related genes analyzed—CDH2, FN1, ZEB1, and SNAI1—only c-Jun was predicted to target them (**Supplementary Table 2**). Following this, we measured the expression levels of VIM, CDH2, FN1, ZEB1, and SNAI1 in H2122 cells after 24 hours of NETs treatment. The findings demonstrated that NETs increased the mRNA expression levels of VIM, CDH2, FN1, and ZEB1 (**Supplementary Figure 6**).

**Figure 6:**
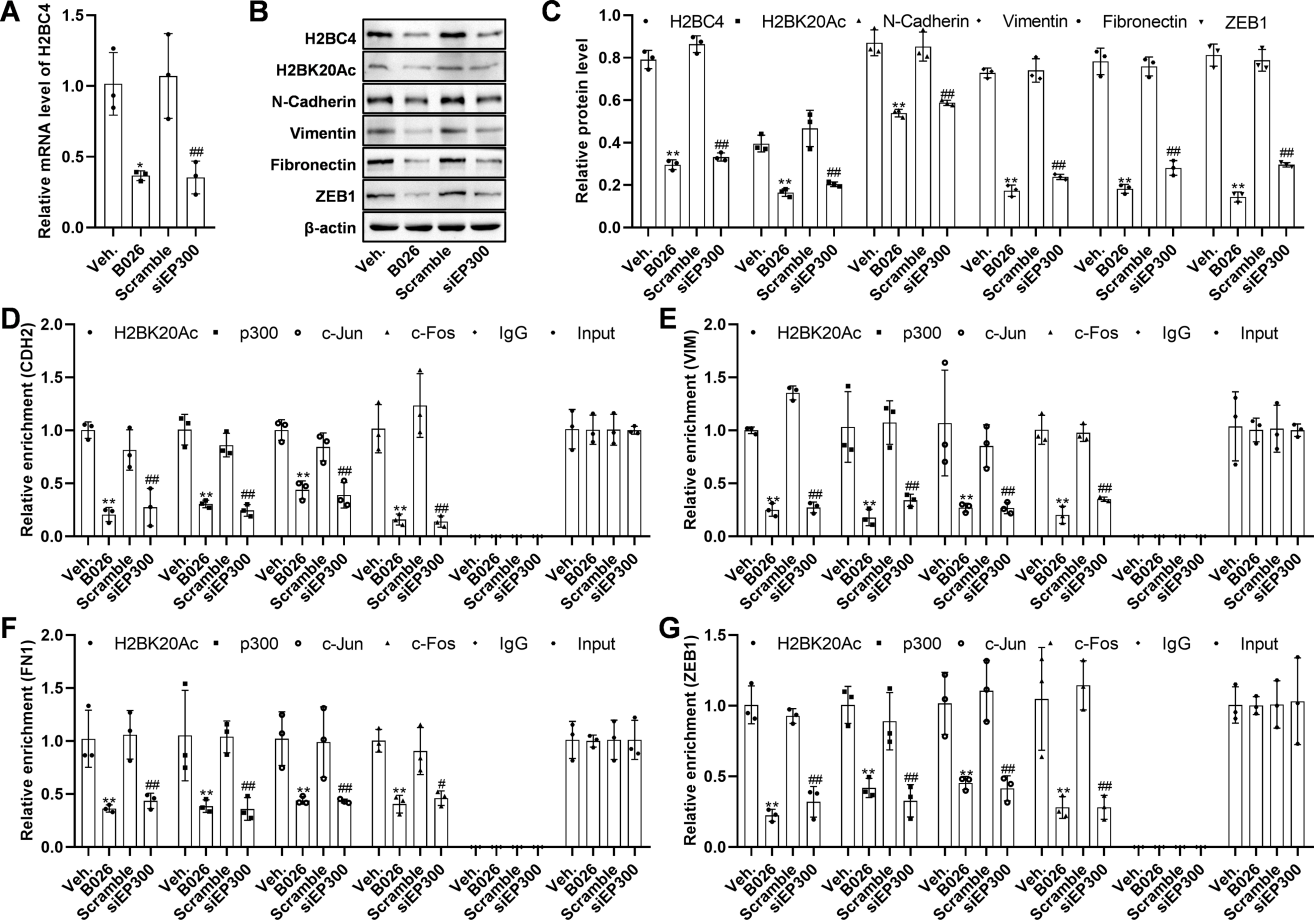
**(A)** the H2BC4 mRNA level was measured in H2122 cells treated with siRNA (siEP300) and small molecule inhibitor B026. **(B, C)**. The levels of H2BC4 protein and H2BC4 acetylation, EMT main marker (N-Cadherin,Vimentin, Fibronectin, and ZEB1) were detected in H2122 cells treated with siRNA (siEP300) and small molecule inhibitor B026. **(D-G)**. ChIP-qPCR examination was used to measure the acetylated H2BC4, EP300, c-Jun, c-Fos enrichment on the promoters of EMT marker genes (CDH2, VIM, FN1, and ZEB1) in H2122 cells treated with NETs. All ChIP enrichment levels are shown relative to Veh. group, and IgG and Input were used as the negative control and positive control, respectively. The experiments were repeated three times (n=3). ^**^P < 0.05, compared with Veh. group; ^##^*P* < 0.01, compared with Scramble group.

To explore the effect of p300 on the acetylation of H2BC4 and on the expression of N-Cadherin, Vimentin, Fibronectin, and ZEB1, we co-treated H2122 cells with EP300 siRNA or the small molecule inhibitor B02 to reduce EP300 expression. EP300 siRNA significantly reduced the mRNA and protein levels, among which siEP300#1 had the highest interference efficiency and will be used as the siEP300 for subsequent experiments (**Supplementary Figures 7A-C**). Cellular thermal shift assay indicated that the small molecule inhibitor B026 reduced the protein level of p300 in a dose-dependent manner (**Supplementary Figures 7A, E**). Compared with NETs alone treating H2122 cells, both siEP300 and the small molecule inhibitor B026 reduced the mRNA level of H2BC4, the protein levels of H2BC4 and H2BK20Ac, and the levels of N-Cadherin, Vimentin, Fibronectin, and ZEB1 (**Figures 6A-C**). Through ChIP-qPCR, we examined the enrichment of acetylated H2BC4, p300, c-Jun, and c-Fos in the promoter regions of EMT marker genes (CDH2, VIM, FN1, and ZEB1) after NETs treatment. The results showed that H2BC4, p300, c-Jun, and c-Fos were enriched in the promoter regions of CDH2, VIM, FN1, and ZEB1.

## 4 Discussion

Neutrophils were initially recognized for their surveillance role and were among the first responders to infection and injury (48). They are the most abundant type of circulating cells in human blood and must activate various defense mechanisms, such as releasing different granular components to ingest, kill, and break down invading pathogens (49). Increasing evidence suggests that NETs have multiple functions in promoting tumors. Similar to histones, NE, and MPO in laboratory settings, NETs components can destroy tumor cells and hinder the growth and spread of tumors (50–52). Research has indicated that various stimuli, including PMA, LPS, cholesterol crystals, ROS formation, and IL-8, can all prompt the formation of NETs (53). Additionally, some studies have demonstrated that IL17 can stimulate neutrophils to migrate to pancreatic tumors, leading to the formation of NETs (54). In our study, we showed that both PMA and IL-17A induced the creation of NETs. There are reports in the literature that IL-17 induces the formation of NETs, such as by facilitating the creation of NETs in patients with oral lichen planus OLP (55).

The presence of NETs can enhance the development and advancement of liver metastasis following surgery-induced stress (56). In mouse models, the formation of NETs and entrapment of circulating lung cancer cells contribute to increased liver micrometastases (21), highlighting the significant role of NETs in facilitating cancer cell metastasis. This research specifically highlights the role of IL-17A in NETs in promoting brain metastasis of lung cancer cells. Although the impact of NETs containing IL-17A on tumor cells has not been observed in other types of cancer, there are reports in literature suggesting its involvement in different diseases. For example, IL17A/F has been detected in NETs in cases of coronary thrombosis following acute myocardial infarction (25). Furthermore, studies indicate that IL-17A-modified NETs can stimulate thromboinflammation and fibrosis in patients with systemic lupus erythematosus (23). Individuals with ankylosing spondylitis demonstrate increased production of NETs carrying bioactive IL-17A, which promote the transformation of bone marrow mesenchymal stem cells into osteoblasts (24). The high concentration of neutrophil-derived IL-17 within the NETs structure implies that its biological function may be enhanced, underscoring the crucial role of the NETs framework in IL-17 activity as seen in various inflammatory conditions (57, 58).

Numerous studies have investigated different elements of NET that enhance the invasion and spread of tumor cells. The process of transfer and diffusion starts with the proteolytic modification of ECM and the release of ECM byproducts. Certain components of NETs play a crucial role in this process, such as NE and MMP-9 derived from NETs, which break down ECM to actively promote tumor invasion (59). The transmembrane protein CCDC25 has been identified as a receptor for NETs-DNA on cancer cells, allowing them to detect extracellular DNA and activate the ILK-β -Parvin pathway to increase cell motility (60). The absence of CCDC25 leads to the elimination of NETs-mediated metastasis. Treatment with DNase I reduces the activities of NE and NETs, as well as diminishes the invasion and metastatic potential of cancer cells (61). The NETs-related MMP-9 in the cerebral microvessels breaks down type IV collagen in the basal layer, disrupting the integrity of the BBB (62). Treatment of MCF7 cells with isolated NETs results in the transformation of epithelial cells into mesenchymal phenotypes, characterized by increased expression of N-cadherin and fibronectin, and decreased expression of e-cadherin, along with enhanced migratory properties (63). Furthermore, in this study, IL-17 presented in NETs induced EMT in lung cancer cells and supports brain metastasis of these cells.

EMT-induced acquisition of mesenchymal phenotypes enhances chemotherapy resistance and leads to a poor prognosis in various cancers (64, 65). In LUAD, NETs are associated with EMT or angiogenesis, weakly linked to the cell cycle, and significantly affect patient prognosis (40). Neutrophils are abundant in gastric cancer and have a negative impact on patient survival by secreting IL-17a and promoting cancer cell EMT through JAK2/STAT3 signaling (43). Several other studies have investigated how NETs affect the EMT of tumor cells. For instance, co-culturing NETs with gastric cancer cells enhanced the expressions of E-cadherin and vimentin, leading to increased cancer cell migration (41). *In vitro* experiments have revealed that colorectal cancer cell lines treated with NETs exhibited filamentous foot formation and enhanced cell motility, triggering EMT in these cells and potentially promoting colorectal cancer metastasis and progression (42). Additionally, NETs have been shown to induce a shift from the typical epithelial morphology to a mesenchymal phenotype in human breast cancer cells (MCF7) by up-regulating the expression of EMT transcription factors like ZEB1 and snail (63). In the context of human pancreatic cancer, NETs have been associated with promoting the migration and invasion of pancreatic cancer cells, as well as facilitating epithelial-mesenchymal transition (66).

Many studies have shown that, along with inducing EMT in tumor cells, the formation of NETs also plays a role in cancer cell metastasis through various mechanisms. Research indicates that in the context of COVID-19, lung inflammation and cytokine storm, together with NETs, can activate dormant cancer cells and create pro-metastasis environments (67). In studies on lung cancer, it has been demonstrated that NETs capture circulating tumor cells and enhance tumor cell metastasis by upregulating NETs and β1-integrin on cancer cells (21, 68). Moreover, NETs protease can break down the extracellular matrix, facilitating cancer cell extravasation and metastasis (13, 51). NETs are also involved in triggering dormant tumor cells to awaken and metastasize (69). NETs can coat and shield tumor cells from CD8^+^ and NK cell infiltration (70). Additionally, NETs can increase blood-brain barrier permeability (71).

Moreover, there is a reciprocal relationship between tumors and NETs. Several studies have confirmed that tumor cells can trigger the formation of NETs through various means, although this particular study did not investigate whether lung cancer cells have the same ability. Metastatic cancer cells can directly trigger the release of NETs without involvement in inflammatory processes (51). In particular, metastatic breast cancer cells can prompt neutrophils to generate NETs, which in turn enhances the proliferation of tumor cells in specific organs (61). In addition, tumor cells have been found to secrete IL-8, attract myeloid-derived suppressor cells, and activate neutrophil precursors to release NETs (72). Furthermore, tumor-derived exosomes from cancer patients experiencing hypercoagulability can stimulate the release of NETs, serving as a platform for coagulation factors, platelets, and exosomes containing pro-thrombotic agents (73). This mechanism collectively promotes the occurrence of thromboembolic complications and advances cancer progression. IL-17A is primarily produced by immune cells (such as Th17, CD8+ T cells, γδ T cells, and NK cells) (74, 75), some studies have also suggested that under certain circumstances, tumor cells—including subtypes of colorectal and breast cancer—may acquire the ability to produce IL-17A, thereby influencing immune cell recruitment and function (76). A study showed that IL-17A/IL-17RC axis enhanced oncogenic signaling and drug resistance in EGFR-mutated LUAD cells (77). Moreover, lung cancer cells can actively induce NET formation by secreting proinflammatory cytokines such as IL-8 (78), which robustly stimulates neutrophil NETs (79). Extracellular RNAs from lung cancer cells have also been shown to activate epithelial cells and promote NET formation (19). Therefore, tumor cells may promote NET formation by secreting cytokines such as IL-17A and IL-8, which in turn establishes a positive feedback loop between tumor cells and neutrophils.

How does IL-17A induce the formation of NETs and how does IL-17A on NETs promote EMT in lung cancer cells and brain metastasis of lung cancer cells? Our research indicates that the levels of both mRNA and protein of H2BC4 are increased in LUAD tumors based on data from the database. Additionally, we observed that the expression and acetylation of H2BC4 rise during the formation of NETs and tumor cell EMT process. IL17A increased the levels of H2BC4, H2BK12Ac, and H2BK20Ac in neutrophils, and NETs also upregulated these the level of molecules in tumor cells. The effects of IL17A and NETs were diminished when treated with anti-IL17A and anti-IL17RA antibodies, suggesting that IL17A/IL17RA signaling is associated with increased expression and acetylation levels of H2BC4 in neutrophils and tumor cells. H2BK20Ac plays a crucial role in acetylation at the K12 and K120 sites of H2B, serving as a unique marker for cell state-specific promoters and enhancers (80). A study has investigated the relationship between H2B acetylation and NETs, revealing that patients with systemic lupus erythematosus have higher levels of acetylated H4-K8,12,16, acetylated H2B-K12, and tri-methylated H3-K27 in NETs compared to healthy individuals (81). In active lupus nephritis, the acetylation of H2B (H2BK12Ac) in microparticles can enhance NET formation (82).

Studies have highlighted the involvement of H2BC4 in various types of cancer. For example, miR-18a-5p, found in high levels in bone metastatic cells in prostate cancer, is transported to the bone microenvironment via EXO where it influences osteoblasts by targeting the H2BC4 gene^81]^. This action leads to increased expression of Ctnnb1 in the Wnt/β-catenin pathway and facilitates the transition of pre-osteoblasts to osteoblasts^81]^. In primary breast tumors, H2BC4 expression is correlated with overall patient survival (83). The expression of H2BC4 differs significantly between tumor cells in triple-negative breast cancer patients and normal breast duct cells. In patients with basal-like subtype 1 breast cancer, the presence of H2BC4 in the primary tumor is linked to survival post-progression (84). Furthermore, the levels of H2BC4 mRNA rise in brain metastatic tissues compared to primary breast tumors (84). The regulation of H2BC4 expression in metastatic breast cancer patients may contribute to the process of breast cancer cells metastasizing to the brain and escaping immune surveillance in lymph nodes (83).

How exactly does the presence of IL17A in IL17A and NETs impact the regulation of H2BC4 expression and acetylation levels in neutrophils and lung cancer cells? There is evidence that the HAT activity of p300 is vital for its function as a co-activator with multiple activators on chromatin (85). Elevated levels of the ubiquitin-26 S proteasome degradation pathway have been observed in differentiated U937 cells, potentially leading to the degradation of p300 (86). The decrease in p300 levels may contribute to a reduction in histone acetylation (86). It appears that p300 plays a key role in the acetylation of H2BC4. Research indicates that IL-6 and IL-17 work together to enhance the acetylation of histones H3 and H4 on the IL-6 promoter, with CBP, p300, and RNA Pol II recruited to initiate IL-6 gene transcription (38). An increase in p300/CBP expression in acute respiratory distress syndrome has been linked to IL-17 (87). Our study reveals that lowering p300 levels results in decreased expression and acetylation of H2BC4 in IL-17A-stimulated neutrophils and NETs-IL-17A-treated tumor cells. This suggests that p300 is probably required for the upregulation of H2BC4 expression and its acetylation in response to IL-17A and NETs-IL-17A. However, whether H2BC4 functionally mediates NETs-IL-17A-induced EMT or brain metastasis requires further investigation, and our current findings support its role as a potential marker of epigenetic changes under these conditions. Based on these findings, while we demonstrate that H2BC4 expression and acetylation consistently correlate with IL-17A and NETs-IL-17A-induced chromatin remodeling and EMT in lung cancer, further studies using selective knockdown or knockout of H2BC4 are needed to establish a direct causal role in metastasis. At present, H2BC4 acetylation can be considered a marker of these processes rather than a proven driver.

This study has several limitations. Our findings primarily rely on *in vitro* and preclinical mouse models, which may not fully recapitulate the complexity of human tumor-microenvironment interactions. The study did not explore potential crosstalk between NETs-associated IL-17A and other immune or stromal cells in the metastatic niche, which could influence therapeutic outcomes. In particular, while mouse and cell-based models provide valuable mechanistic insights, they do not encompass the full diversity and dynamic interplay among cell types found in human tumors, such as distinct immune cell populations, fibroblasts, endothelial cells, and context-dependent molecular signaling. This is an important consideration, as the collective influence of these cells in the metastatic microenvironment may alter or modulate the effect of the NETs/IL-17A axis on tumor progression. Moreover, species-specific differences in immune responses and the lack of human stromal elements in mouse models may limit the direct translatability of our findings to human disease. Future research efforts employing patient-derived xenografts, humanized mouse models, and advanced co-culture or organoid systems, along with integrated single-cell and spatial analysis, will be necessary to delineate the true impact and therapeutic potential of targeting the NETs/IL-17A pathway in human lung adenocarcinoma. While p300 inhibition attenuated metastasis, its systemic effects on normal tissue homeostasis remain to be investigated. Future studies should address these gaps to facilitate translational applications. Our study possibility that NET-associated IL-17A may interact with IL-17RA on tumor cells, providing supportive evidence for a physical or spatial association between the ligand presented on NETs and its receptor on cancer cells. Besides, at the beginning, we thought there might be difference in activity between NETs-IL-17 and free IL-17 like ARG1, which is also found on NETs and cleaved by CTSS into different molecular forms endowed with enhanced enzymatic activity (88). However, the soluble IL-17 and NETs-IL-17 caused comparable expression level change of H2BC4 and its acetylated forms, suggesting no similarity compared with ARG1. However, functional distinction would require additional experiments directly comparing the impacts of NET-bound IL-17A versus soluble IL-17A—such as using NETs with or without IL-17A, or blocking the interaction between NETs-associated IL-17A and tumor cell IL-17RA.

## 5 Conclusion

This study reveals a critical pathway by which IL-17A promotes NETs formation and NETs-associated IL-17A facilitates brain metastasis in LUAD through acetylation modulation of H2BC4. This study not only expands the understanding of NETs in tumor metastasis but also highlights IL-17A/IL-17RA as potential therapeutic targets to disrupt the pro-brain metastasis microenvironment in LUAD.

## Data Availability

The original contributions presented in the study are included in the article/**Supplementary Material**. Further inquiries can be directed to the corresponding author/s.

## Supplementary material

Supplementary Figure 1 The mRNA and protein levels of HPSE in clinical samples of the database. The mRNA data of HPSE obtained from TCGA and CPTAC, clinical proteomic tumor analysis consortium (https://proteomics.cancer.gov/programs/cptac). The protein expression map of HPSE was derived from cProSite (https://cprosite.ccr.cancer.gov/).

Supplementary Figure 2 The H2BC4 gene exhibited strong specificity in neutrophils. **(A)** Expression levels of H2BC4 in various cells obtained from the HPA dataset. **(B)** Expression levels of H2BC4 in various cells obtained from the Monaco dataset.

Supplementary Figure 3 The data from the TCGA database illustrated correlations in expression among the IL17A, IL17RA, H2BC4, and EP300 genes in LUAD.

Supplementary Figure 4 Verification of the interference effect of EP300 siRNA and small molecule inhibitor B026 on p300 in neutrophils. **(A)** RT-qPCR was used to detect the mRNA level of EP300 in neutrophils after siRNA interference. **(B, C)** Western blot was used to detect the protein level of p300 in neutrophils after siRNA interference. **(D, E)**. CETSA, Cellular thermal shift assay was used to detect the inhibitory effect of B026 on p300. The experiments were repeated three times (n=3). ^**^*P* < 0.05, compared with Scramble group; ^##^*P* < 0.01, compared with Veh. group.

Supplementary Figure 5 The transcriptome sequencing results of H2122 cells after treatment with NETs to identify DEGs, differentially expressed genes. **(A)** DEGs were defined based on Padj (<0.05) and |Log2foldchange| >1. **(B)** A total of 1612 DEGs were analyzed using GO, Gene Ontology categories, including biological process, cellular component, and molecular function.

Supplementary Figure 6 After 24 hours of NETs treatment on H2122 cells, the expression levels of CDH2, VIM, FN1, SNAI1, and ZEB1 genes were measured. The experiments were repeated three times (n=3). ^*^*P* < 0.01, compared with control group; ^**^*P* < 0.05, compared with control group.

Supplementary Figure 7 Verification of the interference effect of EP300 siRNA and small molecule inhibitor B026 on p300 in H2122 cells. **(A)** RT-qPCR was used to detect the mRNA level of P300 in H2122 cells after siRNA interference. **(B, C)** Western blot was used to detect the protein level of p300 in H2122 cells after siRNA interference. **(D, E)** CETSA, Cellular thermal shift assay was used to detect the inhibitory effect of B026 on EP300. The experiments were repeated three times (n=3). ^**^*P* < 0.05, compared with Scramble group; ^##^*P* < 0.01, compared with Veh. group.
