# Supplementary figures and images for: Mechanistic and functional characterization of NETs/IL-17 as a therapeutic target in EMT and brain metastasis of lung adenocarcinoma
Source: Front Immunol. 2026 May 25;17:1743841. doi: 10.3389/fimmu.2026.1743841 (PMC13243260; doi:10.3389/fimmu.2026.1743841)

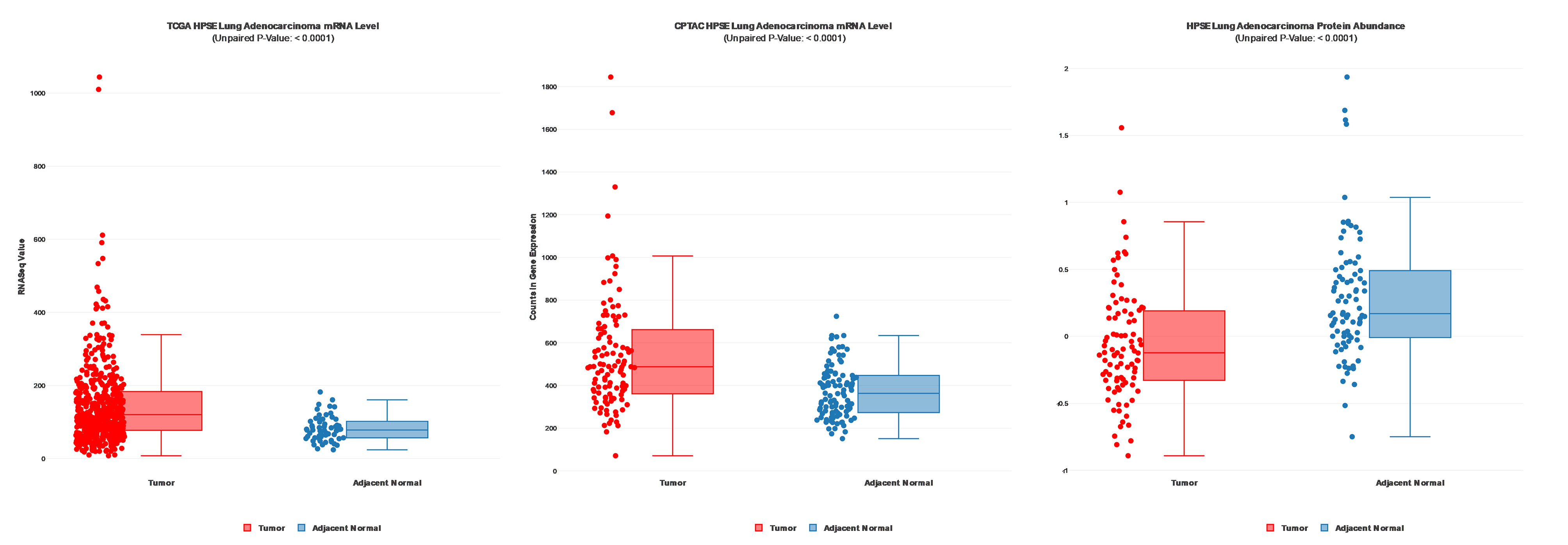

Supplement: Supplementary Figure 1 — The mRNA and protein levels of HPSE in clinical samples of the database. The mRNA data of HPSE obtained from TCGA and CPTAC, clinical proteomic tumor analysis consortium (https://proteomics.cancer.gov/programs/cptac). The protein expression map of HPSE was derived from cProSite (https://cprosite.ccr.cancer.gov/). [file Image1.tif]

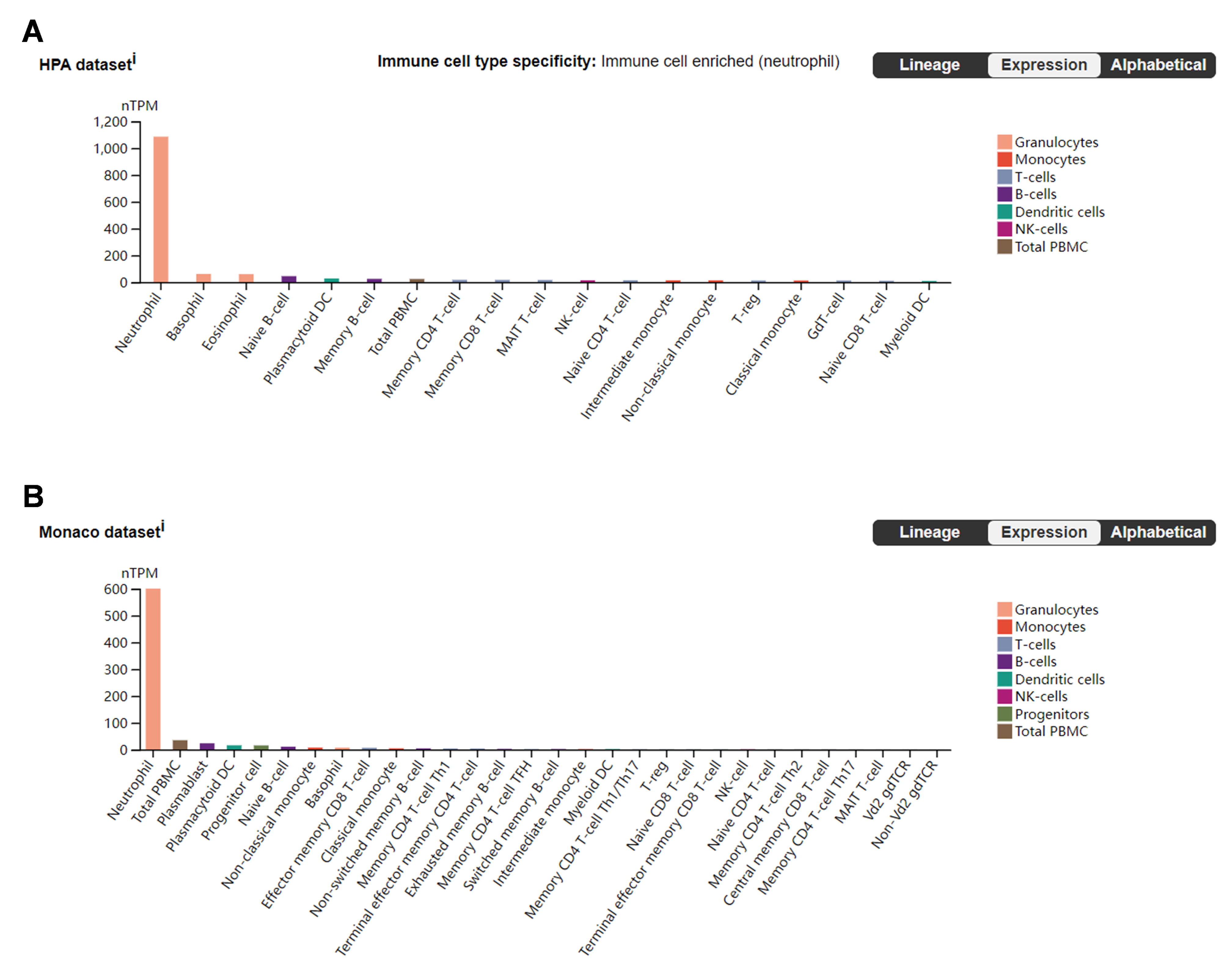

Supplement: Supplementary Figure 2 — The H2BC4 gene exhibited strong specificity in neutrophils. (A) Expression levels of H2BC4 in various cells obtained from the HPA dataset. (B) Expression levels of H2BC4 in various cells obtained from the Monaco dataset. [file Image2.tif]

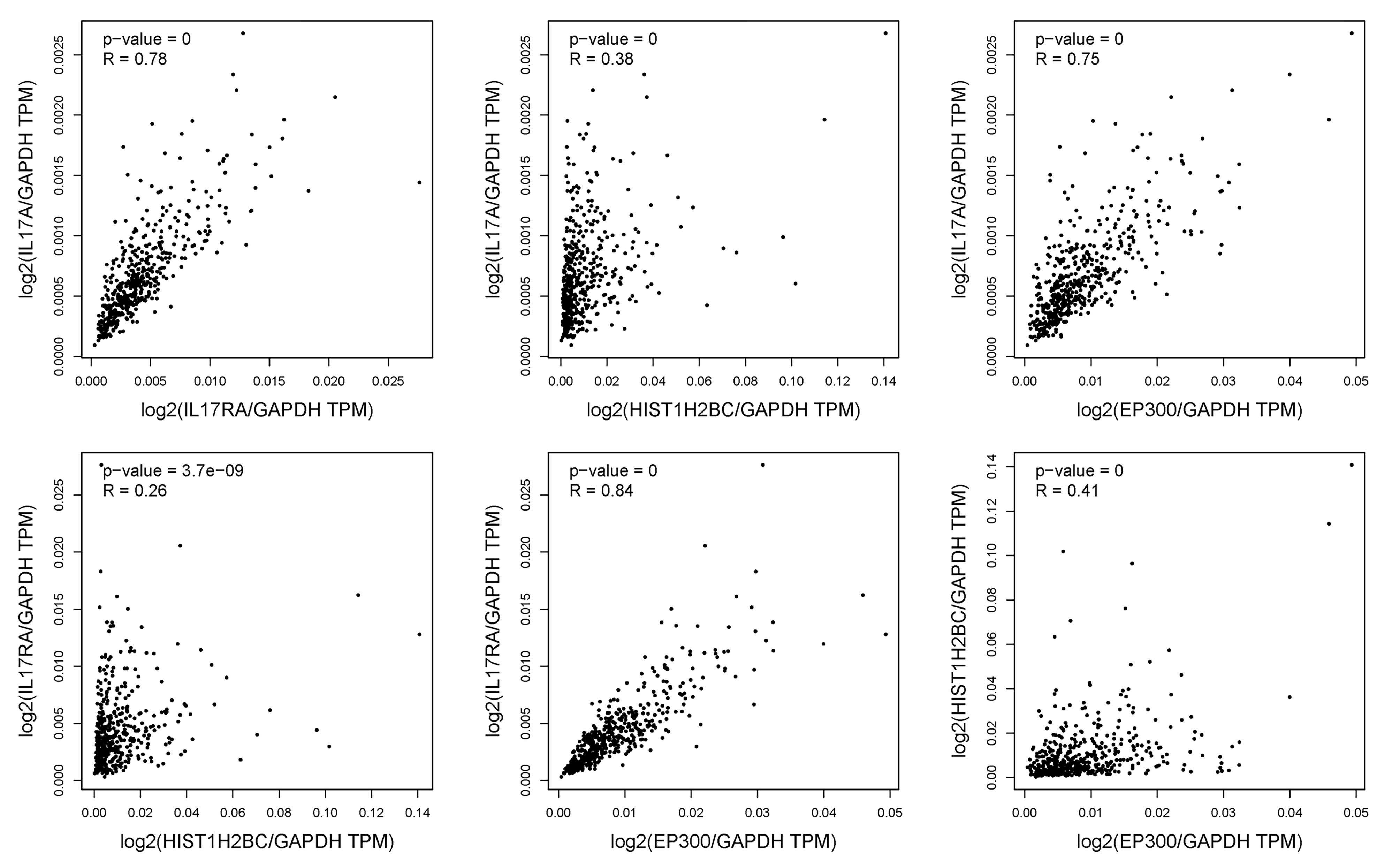

Supplement: Supplementary Figure 3 — The data from the TCGA database illustrated correlations in expression among the IL17A, IL17RA, H2BC4, and EP300 genes in LUAD. [file Image3.tif]

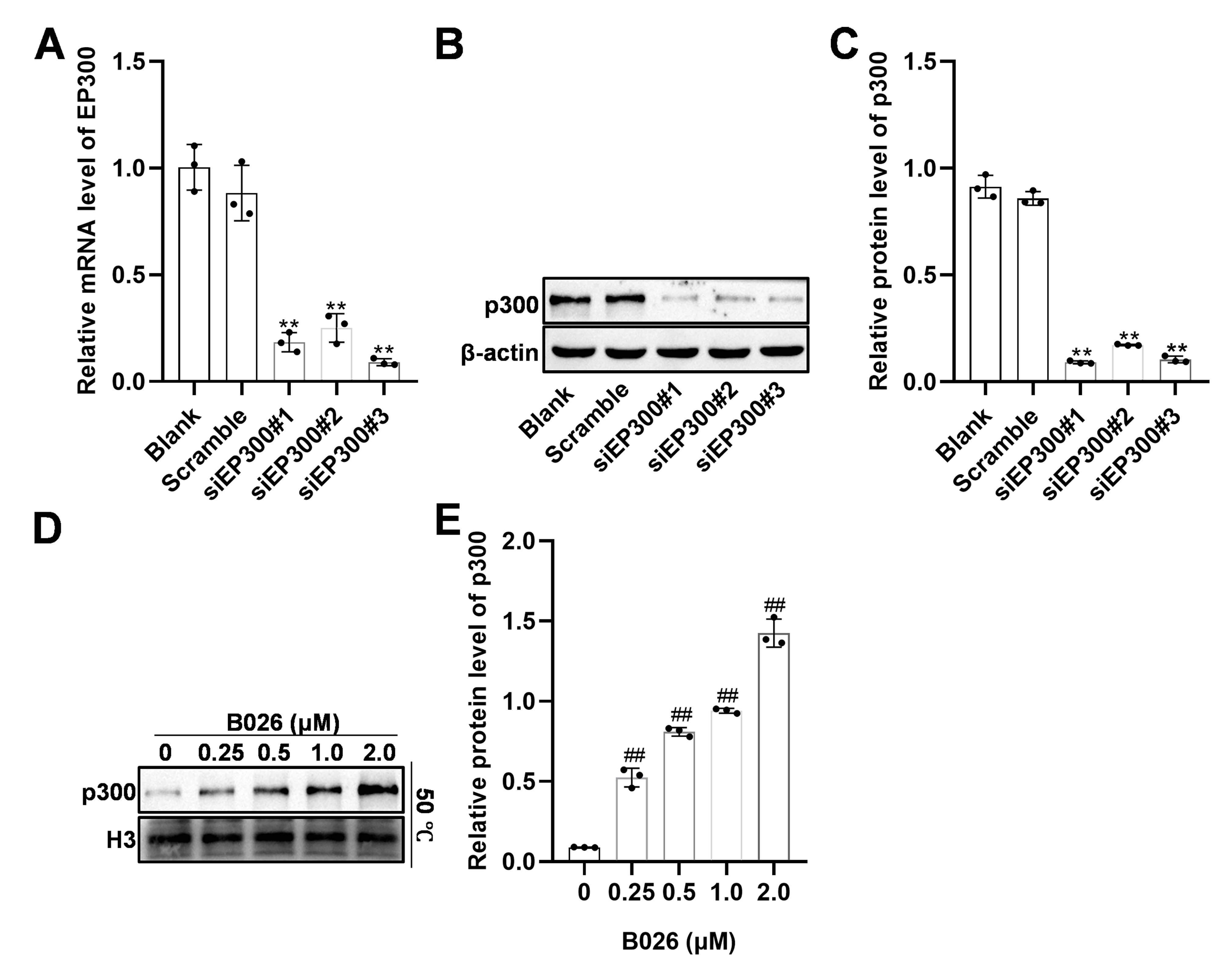

Supplement: Supplementary Figure 4 — Verification of the interference effect of EP300 siRNA and small molecule inhibitor B026 on p300 in neutrophils. (A) RT-qPCR was used to detect the mRNA level of EP300 in neutrophils after siRNA interference. (B, C) Western blot was used to detect the protein level of p300 in neutrophils after siRNA interference. (D, E). CETSA, Cellular thermal shift assay was used to detect the inhibitory effect of B026 on p300. The experiments were repeated three times (n=3). **P < 0.05, compared with Scramble group; ##P < 0.01, compared with Veh. group. [file Image4.tif]

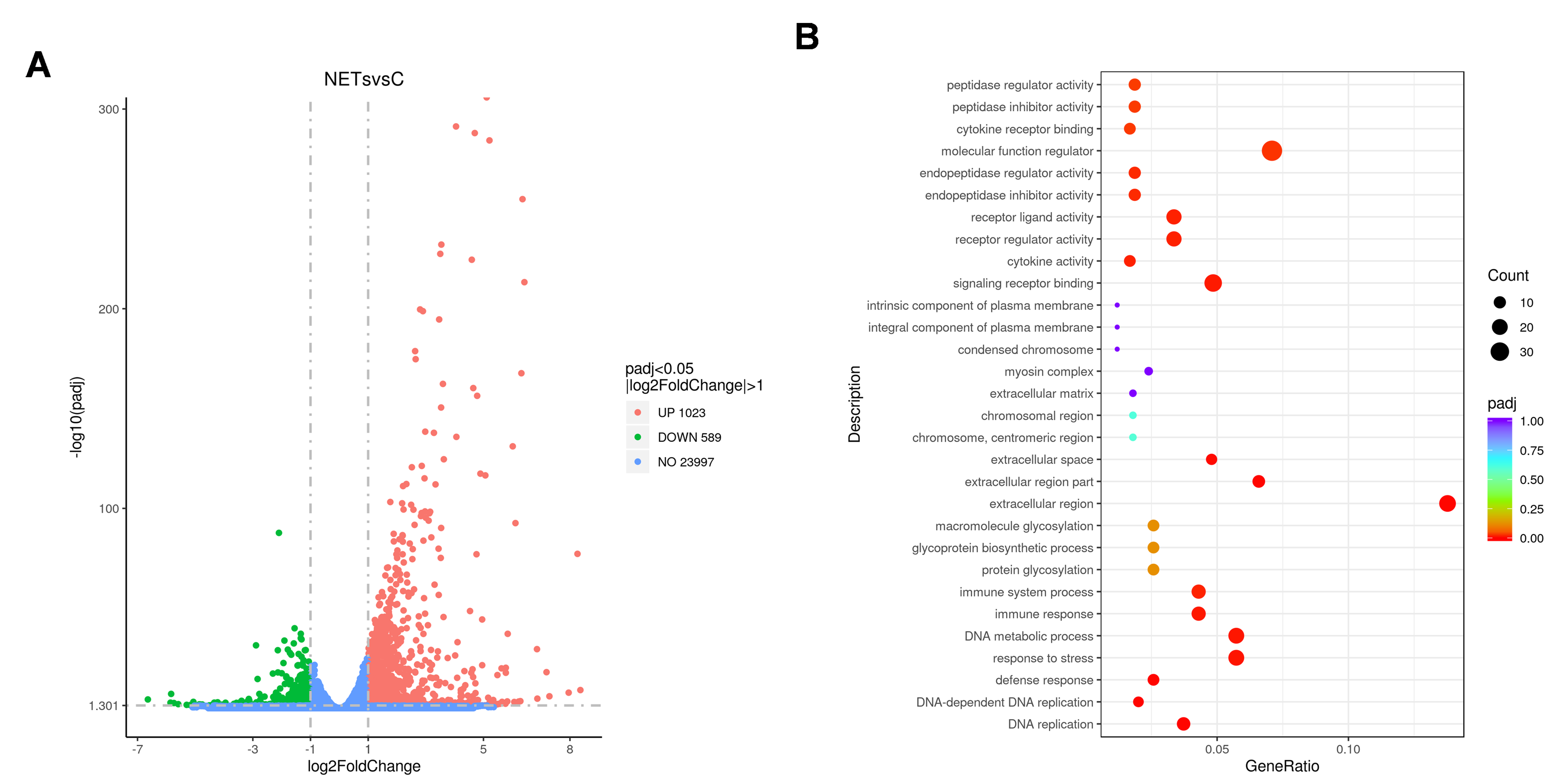

Supplement: Supplementary Figure 5 — The transcriptome sequencing results of H2122 cells after treatment with NETs to identify DEGs, differentially expressed genes. (A) DEGs were defined based on Padj (<0.05) and |Log2foldchange| >1. (B) A total of 1612 DEGs were analyzed using GO, Gene Ontology categories, including biological process, cellular component, and molecular function. [file Image5.tif]

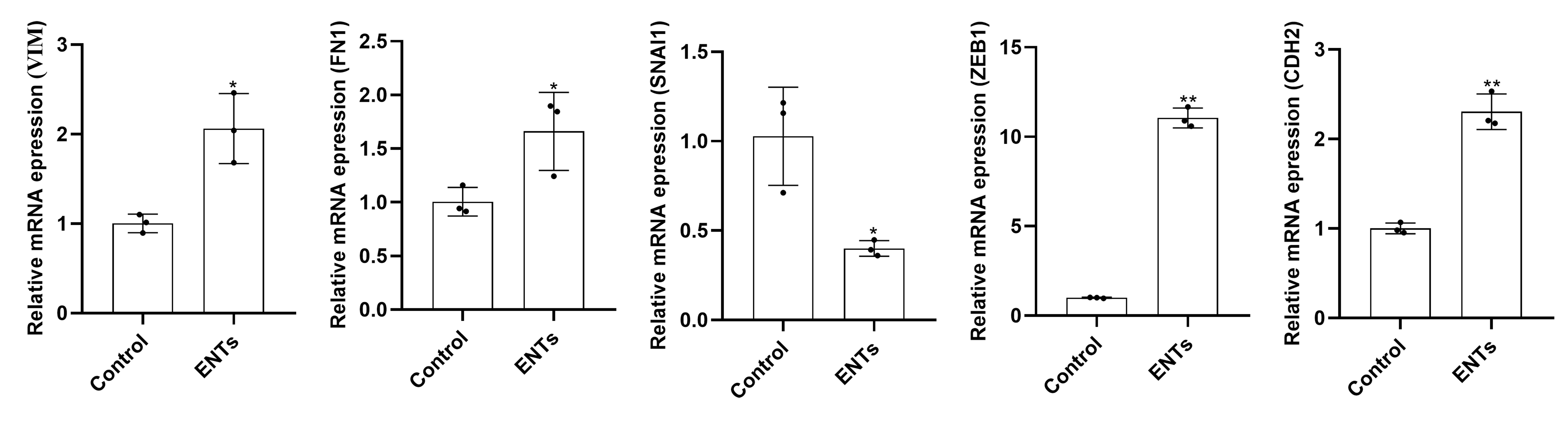

Supplement: Supplementary Figure 6 — After 24 hours of NETs treatment on H2122 cells, the expression levels of CDH2, VIM, FN1, SNAI1, and ZEB1 genes were measured. The experiments were repeated three times (n=3). *P < 0.01, compared with control group; **P < 0.05, compared with control group. [file Image6.tif]

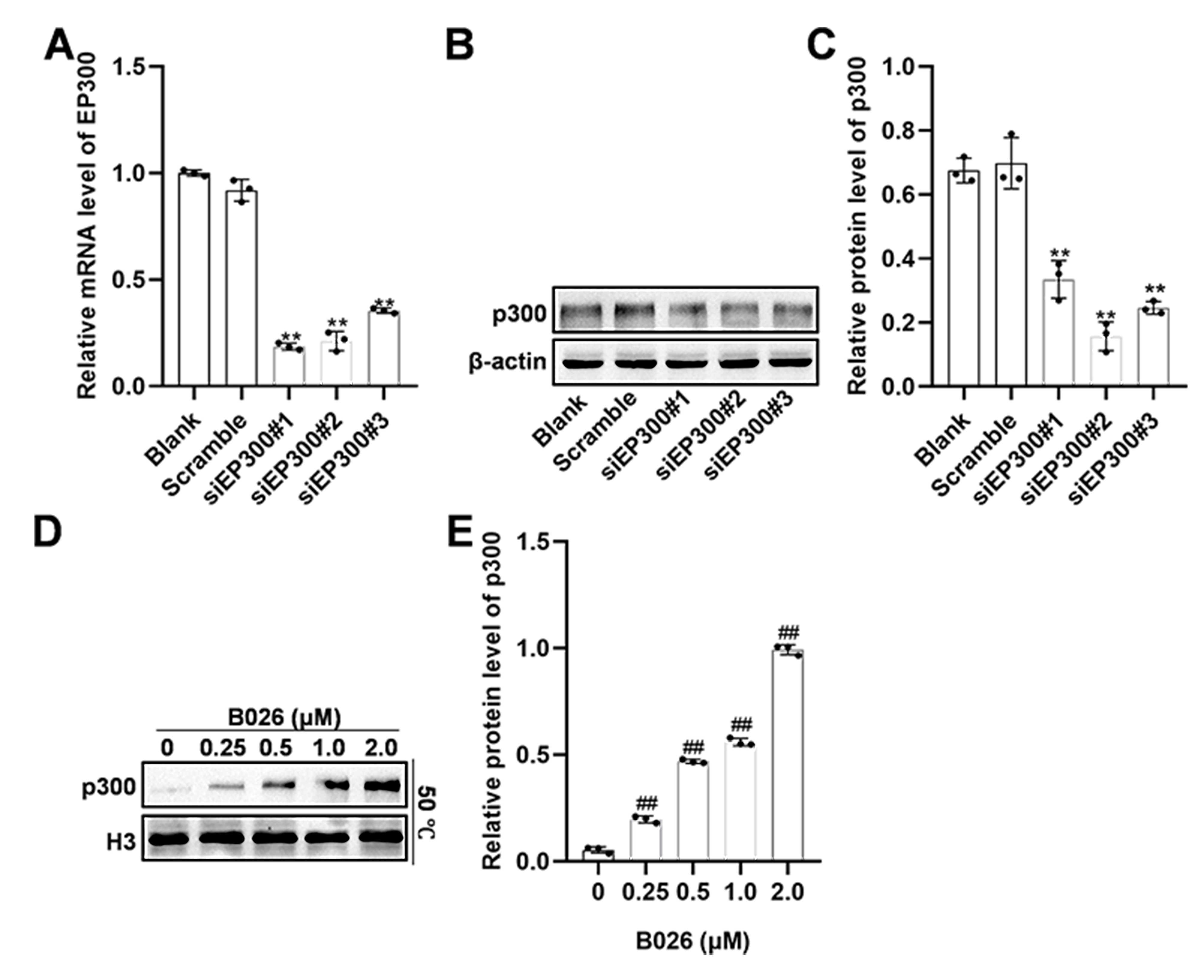

Supplement: Supplementary Figure 7 — Verification of the interference effect of EP300 siRNA and small molecule inhibitor B026 on p300 in H2122 cells. (A) RT-qPCR was used to detect the mRNA level of P300 in H2122 cells after siRNA interference. (B, C) Western blot was used to detect the protein level of p300 in H2122 cells after siRNA interference. (D, E) CETSA, Cellular thermal shift assay was used to detect the inhibitory effect of B026 on EP300. The experiments were repeated three times (n=3). **P < 0.05, compared with Scramble group; ##P < 0.01, compared with Veh. group. [file Image7.tif]

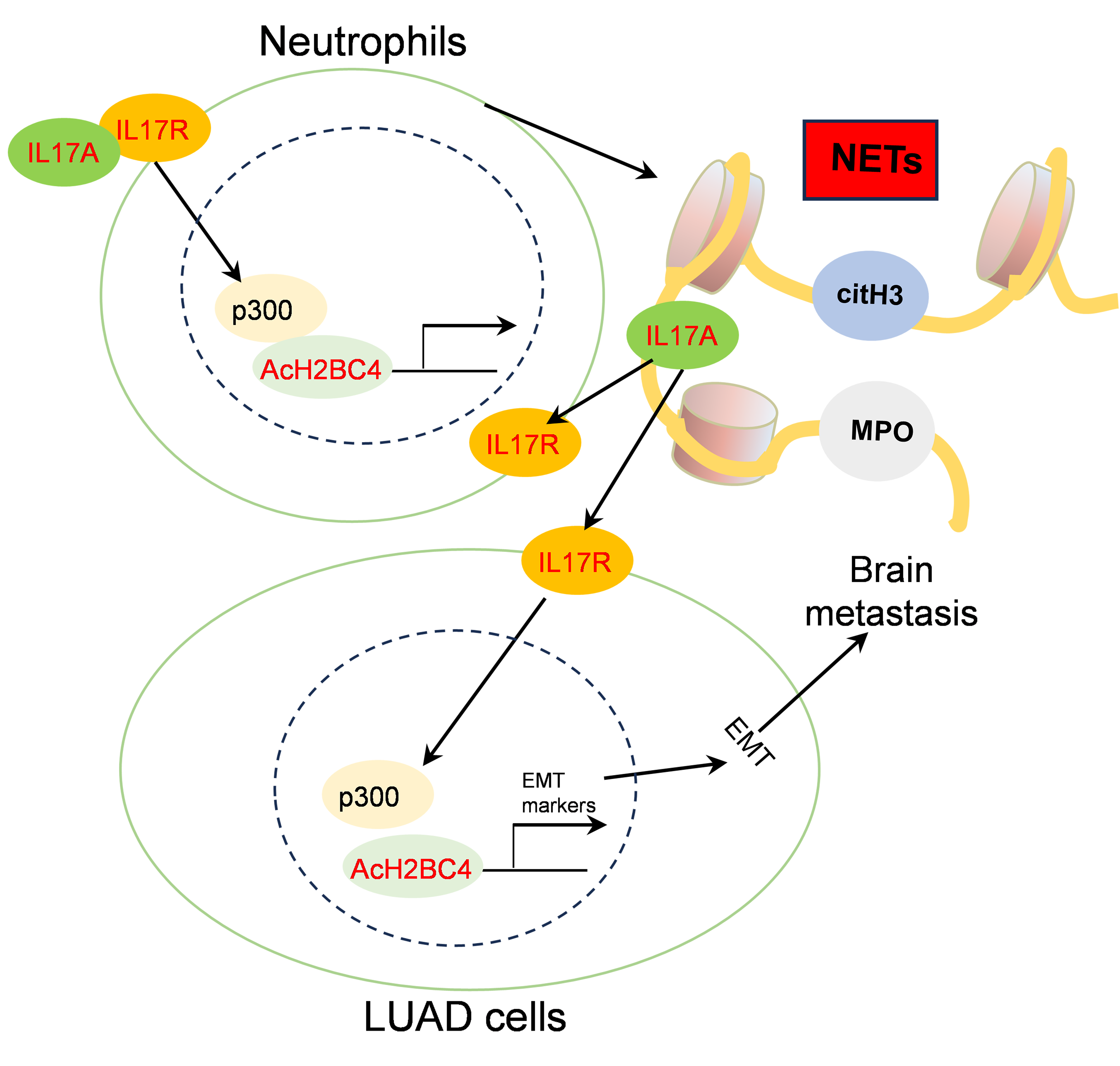

Supplement: Supplementary file 8 [file Image8.tif]
