# Supplementary material for: Mechanistic and functional characterization of NETs/IL-17 as a therapeutic target in EMT and brain metastasis of lung adenocarcinoma
Source: Front Immunol. 2026 May 25;17:1743841. doi: 10.3389/fimmu.2026.1743841 (PMC13243260; doi:10.3389/fimmu.2026.1743841)
Supplement: Supplementary file 9 [file Table1.docx]

**Table S1. List of primer sequences for RT-qPCR analysis**

|  | **Primers** | **Sequence (5’→3’)** |
| --- | --- | --- |
|  | H2BC4-F | GACACTGGCATCTCTTCCAAGG |
|  | H2BC4-R | AGGTGATGGTCGAGCGCTTGTT |
|  | EP300-F | GCAGTGTGCCAAACCAGATG |
|  | EP300-R | GGGTTTGCCGGGGTACAATA |
|  | Vimentin-F | CTTGACGGAAGACATAGTACACC |
|  | Vimentin-R | ACATTGGCAGGAAATAGTCGC |
|  | Fibronectin-F | CCGCCGAATGTAGGACAAGA |
|  | Fibronectin-R | GACAGAGTTGCCCACGGTAA |
|  | ZEB1-F | GTGAAGGTGTACCAGAGGATG |
|  | ZEB1-R | TCTTGCCCTTCCTTTCTGTCA |
|  | CDH2-F | AGCCAACCTTAACTGAGGAGT |
|  | CDH2-R | GGCAAGTTGATTGGAGGGATG |
|  | ACTB-F: | GGCGGCACCACCATGTACCCT |
|  | ACTB-R | AGGGGCCGGACTCGTCATACT |
