# Supplementary material for: Mechanistic and functional characterization of NETs/IL-17 as a therapeutic target in EMT and brain metastasis of lung adenocarcinoma
Source: Front Immunol. 2026 May 25;17:1743841. doi: 10.3389/fimmu.2026.1743841 (PMC13243260; doi:10.3389/fimmu.2026.1743841)
Supplement: Supplementary file 10 [file Table2.docx]

**Supplementary Table 2**. The hTFtarget database was utilized to predict the key EMT-related genes targeted by c-Jun and c-Fos.

| **Protein name** | **Gene name** | **c-Jun** | **c-Fos** | **The impact on EMT** |
| --- | --- | --- | --- | --- |
| Claudin 1 | CLDN1 | × | × | Inhibition |
| EPCAM | EPCAM | √ | √ | Inhibition |
| E-cadherin | CDH1 | √ | × | Inhibition |
| N-Cadherin | CDH2 | √ | × | Promotion |
| Vimentin | VIM | √ | √ | Promotion |
| Fibronectin | FN1 | √ | × | Promotion |
| ZEB1 | ZEB1 | √ | × | Promotion |
| Slug | SNAI2 | × | × | Promotion |
| Snail | SNAI1 | √ | × | Promotion |
| SMAD2 | SMAD2 | × | × | Promotion |
